# Platinum surface oxides govern the cathodic overpotential of the oxygen reduction reaction

**DOI:** 10.1039/d6ey00014b

**Published:** 2026-03-18

**Authors:** Alfred Larsson, Andrea Grespi, Ozbej Vodeb, Karen van den Akker, Auden Ti, Claire Berschauer, Alexandra M. Imre, Philip Miguel Kofoed, Estephania Lira, Mahesh Ramakrishnan, Stuart Ansell, Justus Just, Henrik Grönbeck, Ulrike Diebold, Edvin Lundgren, Lindsay R. Merte, Dusan Strmcnik, Rik Mom, Marc T. M. Koper

**Affiliations:** a Leiden Institute of Chemistry, Leiden University PO Box 9502 2300 RA Leiden The Netherlands m.koper@lic.leidenuniv.nl; b Division of Synchrotron Radiation Research, Lund University Lund Sweden alfred.larsson@fysik.lu.se; c Department of Materials Chemistry, National Institute of Chemistry Ljubljana Slovenia; d Jozef Stefan International Postgraduate School Jamova cesta 39 1000 Ljubljana Slovenia; e Wallenberg Material Science Initiative for Sustainability, Division of Synchrotron Radiation Research, Lund University Lund Sweden; f Institute of Applied Physics, TU Wien Vienna Austria; g Materials Science and Applied Mathematics, Malmö University Malmö Sweden; h MAX IV Laboratory, Lund University Lund Sweden; i Department of Physics and Competence Centre for Catalysis, Chalmers University of Technology Göteborg Sweden

## Abstract

The oxygen reduction reaction (ORR) on platinum is limited by a substantial overpotential, which hampers the efficiency of fuel cell technologies. While adsorbate binding energies have been widely used to explain ORR kinetics, we here illustrate a more complex role of platinum surface oxides, which are often ambiguously defined in the literature. We use *operando* total reflection X-ray absorption fine structure spectroscopy (RefleXAFS), supported by X-ray photoelectron spectroscopy, density functional theory, and microkinetic modeling, to resolve the surface oxides on polycrystalline platinum and their impact on ORR. We identify the formation of a surface oxide as early as 1 V_RHE_ in 0.1 M HClO_4_ and demonstrate that platinum spontaneously oxidizes at the open-circuit potential (OCP) under O_2_ saturation. Furthermore, we show that the oxide coverage increases with upper vertex potential, slower scan rates, and extended hold times at OCP, illustrating how oxides inhibit ORR during fuel cell start-up. Crucially, we demonstrate that the ORR onset is delayed until these oxides are reduced, establishing a direct, negative relationship between oxide coverage and ORR activity. This reveals a revised mechanism in which the potential-determining step is the reduction of surface oxides, and the slow kinetics of this restructuring ultimately determine when surface sites become catalytically available.

## Abstract 2

The oxygen reduction reaction (ORR) on platinum is limited by a substantial overpotential, which hampers the efficiency of fuel cell technologies.

## Graphical abstract

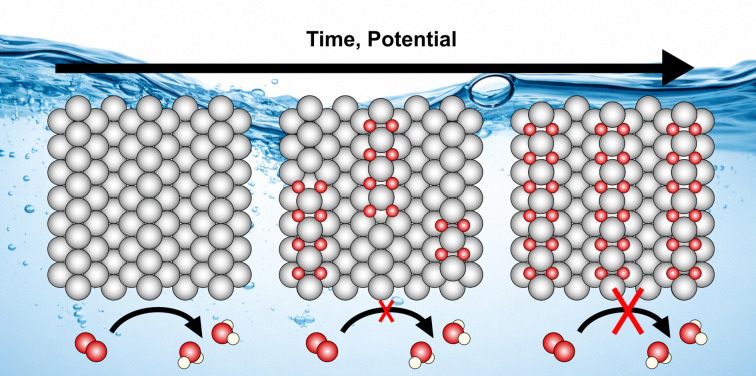

**Broader context**

Fuel cells are a key electrochemical energy conversion technology for a low-carbon energy system, offering zero local emissions when powered by sustainable fuels. Reducing the overpotential of the oxygen reduction reaction (ORR) on platinum, the most active elemental catalyst for ORR, is critical for improving the efficiency and cost-effectiveness of fuel cell technologies. Traditionally, ORR activity has been interpreted through adsorbate-based descriptors derived from idealized model surfaces, an approach that has guided catalyst design for decades. However, realistic fuel cell electrodes operate under dynamic conditions where surface oxidation, restructuring, and transient states are unavoidable. By directly observing platinum surface oxides in *operando* and identifying oxide reduction as the potential-determining step for ORR in the cathodic sweep, this work reveals a previously underappreciated mechanism that governs ORR performance under practical conditions. The finding that platinum oxidizes spontaneously at open-circuit potential further highlights the importance of start-up and shut-down processes in fuel cell operation. Beyond ORR, the methodology and concepts introduced here emphasize the need to consider dynamic surface transformations when designing electrocatalysts, offering a framework applicable to a wide range of energy conversion reactions involving oxide formation and reduction.

## Introduction

The electrochemical oxygen reduction reaction (ORR) plays a pivotal role in fuel cell technologies, where it determines energy efficiency and overall performance.^1–6^ Despite being the most active elemental catalyst for ORR,^7^ platinum exhibits a substantial overpotential,^8^ significantly limiting fuel cell efficiency and hindering widespread commercialization.^9^

Several mechanistic frameworks based on single-crystal studies have been proposed to rationalize the origin of this overpotential on platinum. Based on density functional theory (DFT), OH* reductive desorption has been proposed as the potential-determining step,^10^ while experimental data has suggested that the reduction of O* to OH* governs the overpotential.^11^ Marković and co-workers developed a site-availability model in which spectator species, such as OH* block active sites; in this picture, OH* removal defines the potential-determining step, while the first electron transfer to O_2_ is the rate-determining step.^12^

In realistic catalysts, however, ORR activity is not only influenced by adsorbate chemistry but also by the formation of platinum surface oxides,^13–16^ as observed for platinum nanoparticles in membrane–electrode assemblies^17–20^ and polycrystalline platinum.^21,22^ For the well-defined Pt(111) single-crystal model system, surface X-ray diffraction has shown that ORR overpotential is dictated by adsorbates rather than oxide formation.^23^ However, such a model system underestimates the role of oxidation of steps, kinks, and defects, which oxidize at lower potentials than the (111) facet,^24–26^ and hence can strongly suppress ORR under fuel-cell-relevant conditions, since oxides in general are less active.^27^ Several kinetic models have attempted to account for this effect by incorporating oxide coverage into ORR rate expressions,^18,28^ yet these treatments rely on indirect electrochemical signatures rather than direct spectroscopic observation.^29^

To directly address this gap, we combine *operando* total reflection X-ray absorption fine structure spectroscopy (RefleXAFS)^30–34^ with X-ray photoelectron spectroscopy (XPS), and DFT, to resolve the structure and role of surface oxides on polycrystalline platinum during ORR. We detect the formation of a surface oxide as early as 1.0 V, which also forms spontaneously at the open-circuit potential (OCP) under O_2_ saturation. We show that the presence of this oxide, whose coverage is modulated by upper vertex potential, scan rate, and hold time at OCP, hinders the ORR onset. Microkinetic modeling supports a mechanism in which the overpotential is governed by the sluggish formation and reduction of platinum oxides, where the removal of the surface oxide is the potential-determining step. These findings position surface oxidation as a key factor in ORR catalysis and offer a revised framework for understanding overpotential in realistic fuel cell environments.

## Results

### Formation and structure of surface oxides on polycrystalline platinum

**Fig. 1:**
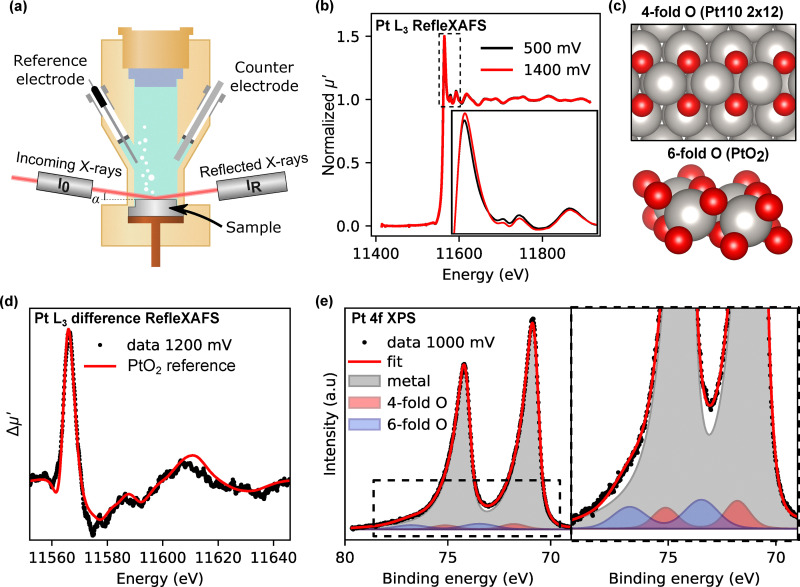
Local structure of platinum surface oxide. (a) Experimental setup for the *operando* RefleXAFS measurement.^34^ (b) Pt L_3_ RefleXAFS spectra at oxidizing (1.4 V) and reducing (0.5 V) conditions measured *operando* in Ar-saturated 0.1 M HClO_4_. (c) Model for the platinum oxide structures with 4-fold oxygen coordination (Pt(110)-(2 × 12)-22O surface oxide along the missing row reconstruction of Pt(110)^35^) and 6-fold oxygen coordinated (PtO_2_). (d) Difference RefleXAFS spectrum (spectrum at 1.2 V subtracted by spectrum at 0.5 V). The red line shows the experimental difference spectrum for PtO_2_ (6-fold O), as in (c). (e) *Ex situ* Pt 4f XPS spectrum after holding at 1.0 V in 0.1 M HClO_4_.

To investigate the formation of surface oxides on polycrystalline platinum under electrochemical conditions, we performed *operando* RefleXAFS measurements using the setup shown in Fig. 1a. This technique enables real-time, surface-sensitive X-ray absorption measurements of planar electrodes under potential control in contact with an electrolyte. However, it is important to note that spectra measured in reflection are distinct from traditional X-ray absorption spectra measured in transmission. This is further described in the SI. Here we will use *µ*′ to denote the absorption coefficient extracted from RefleXAFS.

Fig. 1b shows normalized Pt L_3_-edge RefleXAFS spectra collected under reducing (0.5 V) and oxidizing (1.4 V) conditions in 0.1 M HClO_4_. The spectral changes are highlighted in the difference spectrum in Fig. 1d, which reveals a pronounced white line feature at ∼11 570 eV, indicative of platinum oxidation, along with distinct fine structure beyond the edge. The spectral signature aligns well with the experimental difference spectrum of bulk PtO_2_ as shown in red (transmission spectrum from a PtO_2_ pellet, where the transmission spectrum from a metallic Pt foil has been subtracted).

To further confirm the presence of the surface oxide and its components, we performed *ex situ* Pt 4f XPS after a 10-minute potential hold at 1.0 V in 0.1 M HClO_4_ (Fig. 1e). The spectrum displays two additional doublets beyond the metallic platinum signal (4f_7/2_ at 71.0 eV). These two doublets were not observed in the spectra after potential holds at 0.5 V (see SI Fig. S18 and S19), indicating that they originate from the oxidation of the surface. One is shifted by 0.8 eV, consistent with DFT-calculated values for 4-fold surface oxides (0.68–0.87 eV described in the SI, Fig. S20 and Table S1), and with previous literature values for surface oxides on platinum with 4-fold oxygen coordination.^25,36^ The second doublet with a core-level shift of 2.5 eV agrees well with literature values for the 6-fold oxygen-coordinated structure of PtO_2_.^14,36^ Structures of 4-fold and 6-fold oxides are shown in Fig. 1c. These results confirm our ability to detect surface oxides on platinum, in an electrochemical environment and under potential control using *operando* RefleXAFS.

Later, we will use the term coverage to quantify the amount or degree of oxidation of the surface. Note that coverage here is more complicated than a quantity between 0 and 1 monolayer. Based on the XPS results, we obtain an oxide thickness of 2.5 Å at 1 V (see the SI for the calculation). This is in the expected range for a monolayer of oxide, but we cannot say whether the oxide follows layer-by-layer growth or if island growth also occurs.

### Initial oxide coverage determines the onset potential for ORR

**Fig. 2:**
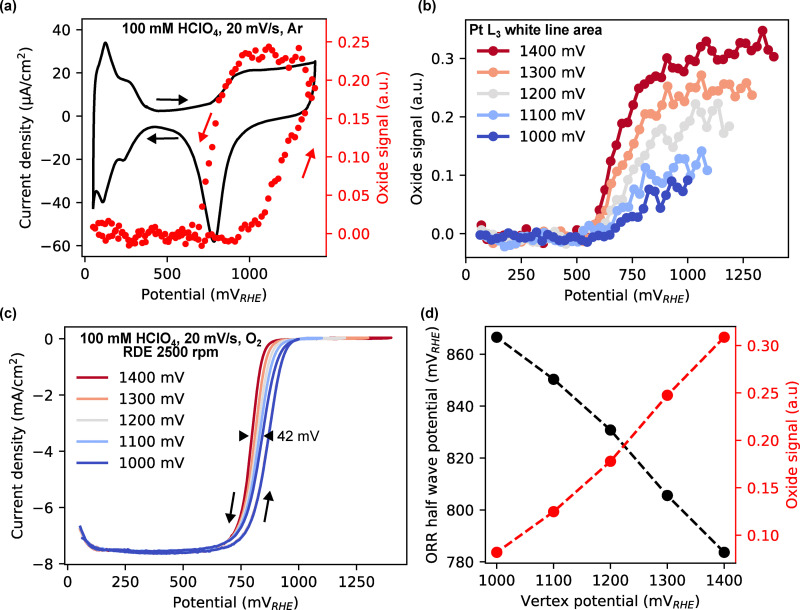
Effect of upper vertex potential on the onset of ORR. (a) (black) CV of polycrystalline platinum in Ar-saturated 0.1 M HClO_4_ measured using the *operando* synchrotron flow cell. (red) Corresponding oxide signal extracted from RefleXAFS. (b) Oxide signal during negative-going sweeps for varying upper vertex potentials. (c) CVs of polycrystalline platinum in Ar-saturated 0.1 M HClO_4_ measured using RDE at 2500 rpm at 20 mV s^−1^. (d) Negative-going ORR half-wave potential and initial oxide signal as a function of upper vertex potential.

To examine how the initial surface oxide coverage affects ORR, we first quantified oxide formation dynamics using RefleXAFS during cyclic voltammetry (CV) in Ar-saturated 0.1 M HClO_4_ at 20 mV s^−1^. Fig. 2a shows the CV alongside the corresponding RefleXAFS oxide signal, extracted by integrating the absolute value of the difference spectra (details in the SI, Fig. S8–S10). During the positive-going scan, oxide formation begins before 1.0 V, highlighting this as the onset potential for surface oxide formation. The oxide signal increases linearly beyond 1.0 V and decreases below ∼0.8 V during the negative-going scan, in agreement with prior studies using XPS, XAS, and EQCM.^14–16,37–39^

To test the influence of oxide coverage on ORR, we conducted negative-going sweeps in an O_2_-saturated electrolyte after 30-second holds at various upper vertex potentials. Fig. 2b shows that increasing the upper vertex potential leads to higher initial oxide coverage (the corresponding electrochemical data are shown in the SI, Fig. S11 and the oxide signal as a function of time during the potential hold at 1 V is shown in Fig. S13). During subsequent negative-going sweeps, this coverage is fully reduced only after reaching potentials below ∼0.6 V. Fig. 2c presents CVs of a similar upper vertex experiment in O_2_-saturated conditions. For higher upper vertex potentials, the onset of ORR shifts to lower potentials, meaning higher overpotentials, consistent with the presence of site-blocking surface oxides. The hysteresis of the oxide signal in Fig. 2a does not match the hysteresis in the ORR current in Fig. 2c, indicating that in the positive-going scan, the role of adsorbates needs to be considered, and in the negative-going scan, the reaction reaches mass-transfer limitation before all of the oxide is reduced. The correlation between initial oxide coverage and ORR onset is quantified in Fig. 2d, which shows the negative-going ORR half-wave potential against the initial oxide signal extracted from RefleXAFS. A clear linear negative relationship is observed: higher oxide coverage results in more negative ORR onset/half-wave potentials. These findings indicate that surface oxides hinder ORR initiation and must be reduced before catalytic activity resumes. This also holds true for adsorbing anions (H_2_SO_4_) and in alkaline conditions, as shown in the SI, Fig. S12 and S13.

### Kinetic control of surface oxidation modulates ORR overpotential

We next investigated how electrochemical protocol parameters, such as scan rate and hold time at OCP, modulate surface oxide coverage and influence ORR.

**Fig. 3:**
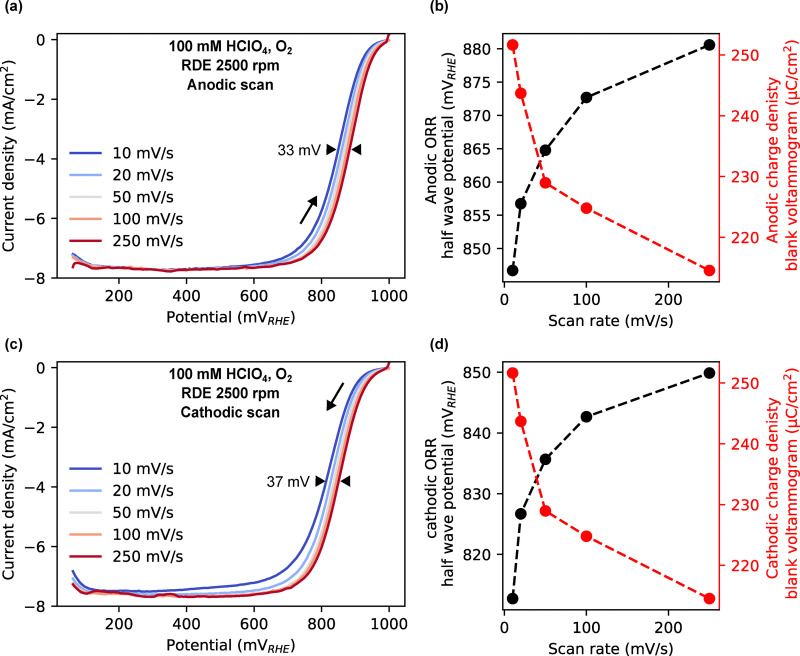
Effect of scan rate on ORR. (a) and (c) Blank subtracted positive and negative-going voltammograms up to 1 V at varying scan rates measured for polycrystalline platinum in O_2_-saturated 0.1 M HClO_4_ at 2500 rpm. (b) and (d) Positive and negative-going ORR half wave potential and anodic and cathodic charge density from blank voltammogram as a function of scan rate.

Fig. 3a and c show CVs up to 1 V separated into positive and negative-going sweeps under O_2_ at scan rates ranging from 10 to 250 mV s^−1^. The voltammetric features of the blank voltammogram (Ar-saturated) have been subtracted to only show the current due to oxygen reduction (see SI Fig. S27). In the positive-going sweep, the ORR half-wave potential shifts positively with increasing scan rate, indicating lower overpotentials, in line with observations in the literature.^40–42^ To quantify this effect, we correlated the positive-going ORR half wave potential with the anodic charge density from the blank voltammograms (shown in SI, Fig. S26) as the oxidation behavior is not affected by the presence of O_2_ (shown in the SI Fig. S16). As shown in Fig. 3b, a direct negative relationship is again observed, confirming that kinetically delaying the formation of oxygenated adsorbates decreases the overpotential for ORR in line with previous observations.^11,43^ In the negative-going sweep, shown in Fig. 3c, the ORR half wave potential also shifts positively with increasing scan rate and is quantified in Fig. 3d. At slower scan rates, the electrode remains longer at oxidizing potentials, resulting in a higher surface oxide coverage and thus explaining the scan-rate dependence observed in the negative-going sweep. When the oxide coverage prior to the negative-going sweep is equalized for all scan rates by introducing a hold at the upper vertex potential, the activity trend is reversed, as shown in the SI, Fig. S29.

**Fig. 4:**
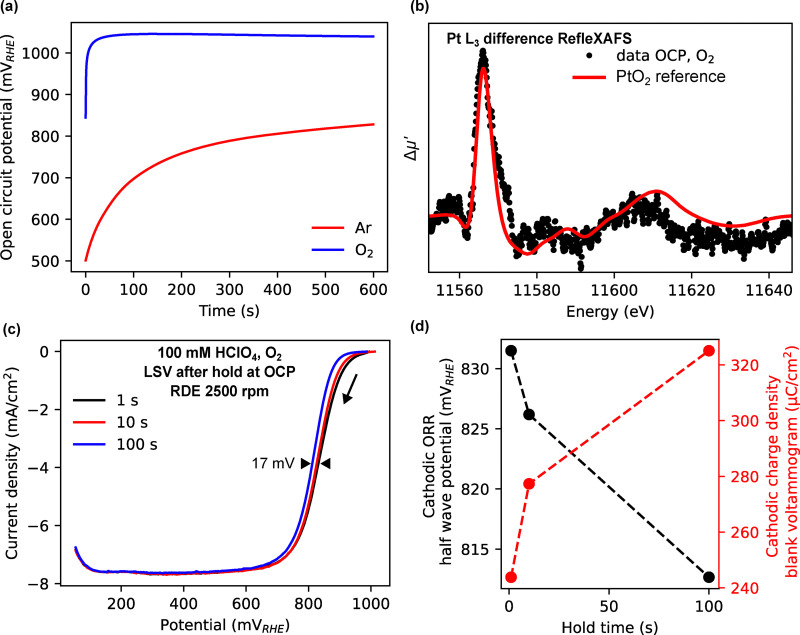
Effect of hold time at OCP on ORR. (a) OCP *vs.* time measured for polycrystalline Pt in 100 mM HClO_4_ in O_2_ and Ar-saturated conditions. (b) Difference RefleXAFS spectrum (spectrum at OCP subtracted by spectrum at 0.5 V). The red line show reference spectra for PtO_2_. (c) LSVs after varying hold time at 1.0 V measured for polycrystalline platinum in O_2_-saturated 0.1 M HClO_4_ at 2500 rpm and 20 mV s^−1^. (d) Negative-going ORR half wave potential and cathodic charge density of the reduction peak of the blank voltammograms as a function of hold time.

We further examined the effect of hold time at the OCP of platinum in O_2_-saturated conditions. Fig. 4a shows that the OCP in O_2_-saturated conditions reaches above 1 V, significantly higher than in Ar-saturated conditions. At OCP, in O_2_ saturated conditions, the potential is high enough to spontaneously oxidize the platinum surface, as shown in Fig. 4b. When the hold time at OCP is varied, the oxide coverage increases, which is reflected in a shift in the onset of ORR as shown in the linear sweep voltammograms (LSV) in Fig. 4b. This is further quantified in Fig. 4c, which shows the negative-going ORR half-wave potential and the integrated charge of the reduction peak in the blank voltammogram after potential holds at 1000 mV (shown in the SI, Fig. S28). Again, a clear negative relationship is observed.

These experiments collectively demonstrate that surface oxide coverage, governed by electrochemical protocol parameters and the spontaneous oxidation at OCP, directly impacts the ORR onset. The data support a mechanism in which oxide formation and reduction kinetics control the overpotential under these conditions.

### Microkinetic modelling of platinum oxidation and ORR onset

**Fig. 5:**
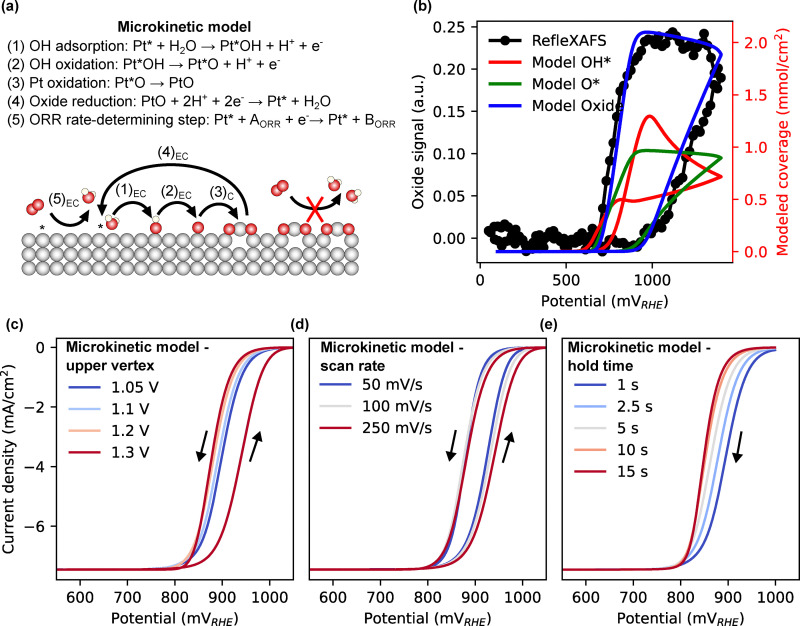
Microkinetic modeling: (a) schematic illustration of the microkinetic model. (b) Experimental oxide signal measured using RefleXAFS at 20 mV s^−1^ and coverages extracted from the microkinetic model at 100 mV s^−1^. (c) Modeled CVs at 250 mV s^−1^ in O_2_-saturated conditions for different upper vertex potentials. (d) Modeled CVs in O_2_-saturated conditions for different scan rates. (e) Modeled negative-going LSVs at 250 mV s^−1^ in O_2_-saturated conditions after different hold times at 1 V.

To further support the conclusion that surface oxide coverage governs ORR onset and overpotential, we developed a microkinetic model using Electrokitty^44^ of platinum oxidation and oxygen reduction (details in SI) parameterized by fitting blank CVs of varying scan rates and upper vertex potentials. The oxidation mechanism, illustrated in Fig. 5a, involves an initial electrochemical adsorption of OH*, followed by its oxidation to O*. The adsorbed O* can then irreversibly transform into a chemically formed PtO species, representing the surface oxide. This oxide can be electrochemically reduced in a single step to regenerate active platinum sites.

Fig. 5b compares the simulated and experimental oxide coverage during cyclic voltammetry, showing good agreement. Simulated blank voltammograms are provided in the SI, Fig. S29–S32. To incorporate ORR, we added a reversible single-electron transfer reaction that occurs exclusively on free platinum sites. Fig. 5c presents simulated CVs under O_2_-saturated conditions with varying upper vertex potentials. As observed experimentally, increasing the vertex potential raises the oxide coverage and shifts the ORR onset to more negative values. Fig. 5d shows the effect of scan rate, where faster scans yield lower overpotentials in both the positive and negative going scans. Fig. 5e shows the effect of hold time, where longer holds result in higher oxide coverage and higher overpotentials.

These simulations qualitatively reproduce the experimental trends, reinforcing the conclusion that ORR overpotential is governed by the formation and reduction kinetics of platinum oxides.

## Discussion

Our results provide new insights into the role of platinum surface oxidation in governing the ORR by combining *operando* spectroscopy, electrochemistry, and microkinetic modeling. A central advance is the application of real-time quick-scanning, surface-sensitive RefleXAFS, which enables direct monitoring of platinum oxide formation and reduction under electrochemical operating conditions. Unlike conventional electrochemical methods, which infer oxidation states from indirect current–potential signatures, RefleXAFS provides spectroscopic evidence of oxide growth and reduction dynamics, resolving long-standing ambiguities about how “oxide” should be defined in the ORR context. This methodological development establishes a pathway for investigating dynamic surface transformations of flat extended electrodes under realistic conditions.

The oxygen reduction reaction is a complex multistep process generally described by the associative pathway involving four proton-electron transfer steps. Based on DFT, the widely adopted model for Pt(111) identifies OH desorption as the potential-determining step.^10^ Liu and Koper, in contrast, proposed that the conversion of O* to OH* governs the overpotential on Pt(111).^11^ An alternative perspective is provided by the site-availability model developed by Marković, which emphasizes the role of spectator species: here, site blocking by OH* controls the apparent activity, with the potential-determining step being OH removal, while the rate-determining step is O_2_ conversion to OOH*.^12^ These models, while insightful, have largely been constructed around Pt(111), a surface dominated by extended terraces that oxidizes only at high potentials.

Our results extend beyond these models by demonstrating that on polycrystalline platinum, oxidation and surface oxide reduction become the key factors controlling activity. We propose that rather than OH* removal or O* conversion, the oxidation and oxide reduction dynamics govern the overpotential for ORR on polycrystalline Pt. To capture this with a site blocking term (1 − *θ*), not only coverage by oxygenated species but also structural rearrangements of the surface need to be considered. During the formation of the surface oxide, platinum atoms migrate to accommodate the new oxide lattice, and the slow kinetics of this restructuring ultimately determine when active surface sites become catalytically available again in the cathodic sweep, where the surface oxide is reduced. On these active sites, the activity should then be governed by the rate-determining step. This means that ORR is not limited by inherently slow kinetics caused by the transition state energy of intermediates, but rather by site availability. This mechanistic picture, consistent across multiple experimental variables (upper vertex potential, scan rate, and hold time), reconciles the discrepancy between Pt(111)-based models, which emphasize adsorbates, and the behavior of more realistic, defect-rich catalysts, where oxidation occurs at steps, kinks, and other low-coordination sites that oxidize at lower potentials.

Surface X-ray diffraction has previously been used to study the oxidation of platinum single crystals, in particular Pt(111),^45^ which exhibits a more reversible behavior of the surface oxidation and reduction compared to polycrystalline platinum studied here. This indicates that O* on Pt(111) is easier to reduce, which may be explained by a different surface oxide structure compared to polycrystalline Pt. It is therefore expected that the delay of ORR due to oxide reduction is less pronounced on Pt(111), given that the upper vertex stays below that of irreversible surface transformation ∼1.2 V.^46^ However, for polycrystalline Pt, since oxidation and reduction cycles have been used to condition the surface, we assume that upper vertex potentials of up to 1.4 V do not lead to further substantial changes of the surface.

Surface oxidation also provides a possible explanation for discrepancies in reported structure–activity relationships. In acidic electrolytes, ORR activity in the anodic sweep increases with step density,^47^ explained by local stress release,^48^ whereas in alkaline electrolytes, activity decreases with step density.^49^ Liu and Koper attributed this to cation blocking of step sites in alkaline media.^11^ An alternative interpretation is that step oxidation occurs within the ORR potential range in alkaline conditions, passivating active sites and reducing activity. In acidic conditions, by contrast, oxidation sets in at higher potentials, delaying passivation and leaving activity governed predominantly by adsorbates.

Finally, we observe that platinum undergoes spontaneous oxidation at OCP, in O_2_-saturated conditions. This observation carries significant implications for fuel cell operation, particularly during start-up and shut-down cycles, where the presence of surface oxides inhibits ORR initiation and contributes to performance losses. Understanding and controlling this spontaneous oxidation process could therefore be critical for improving fuel cell durability and efficiency.

Taken together, our findings establish a revised framework for ORR on platinum surfaces. Adsorbate-based descriptors remain appropriate for Pt(111), and for the positive-going sweep on polycrystalline Pt, but in the negative-going sweep of realistic catalysts, the overpotential is dictated by the slow kinetics of oxide formation and reduction caused by structural rearrangements that are distinctly different from adsorption processes. Surface oxidation does not affect the ORR activity during steady state operation of fuel cells in the mass-transfer-limited regime once all the surface oxides are reduced. However, this has significant implications for fuel cell start-up and shut-down as well as for theoretical studies, which typically assume clean, unreconstructed surfaces and neglect the role of surface oxides.^12,50–55^

## Conclusions

This work establishes that platinum surface oxidation, rather than solely adsorbate energetics, is the dominant factor controlling ORR on polycrystalline platinum. Using *operando* RefleXAFS, complemented by XPS, DFT, and microkinetic modelling, we directly resolve the structure and dynamics of platinum oxides under electrochemical conditions. We identify surface oxides that form already at 1 V and even spontaneously at open circuit potential in O_2_-saturated environments. Their coverage increases with upper vertex potential, slower scan rates, and longer OCP holds, and we show a direct, negative correlation between oxide coverage and ORR onset.

Beyond these observations, our data deliver an important mechanistic advance. Whereas classical ORR models emphasize elementary proton–electron transfers and site blocking by OH*, we demonstrate that the key potential-determining step on realistic, defect-rich platinum surfaces is the reduction of surface oxides. The sluggish restructuring required to remove these oxides delays the unblocking of catalytically active sites, and thereby sets the effective ORR overpotential where the removal of the surface oxide is the potential-determining step. This framework bridges the terrace-dominated Pt(111) studies, where adsorbate binding energies are decisive, and practical catalysts, where steps and defects oxidize within the ORR potential window.

Altogether, these results position platinum oxidation as a central mechanistic feature of the ORR, shifting the focus from static binding energy descriptors toward the dynamic formation and reduction of oxides. This insight has direct implications for fuel cell operation, particularly during start-up and shut-down, where spontaneous oxidation at OCP inhibits ORR initiation. Strategies that mitigate oxide formation or accelerate oxide reduction—whether by alloying, surface modification, or operational control—may therefore be essential for practically overcoming the persistent ORR overpotential on platinum catalysts.

## Methods

### Chemicals

The following chemicals were used for the cleaning procedure without further pretreatment: K_2_MnO_4_ (Emsure, ≥99.0%), H_2_SO_4_ (Sigma Aldrich 95.0–97.0%), and H_2_O_2_ (Merck, 35%). The following chemicals were used without further pretreatment to mix electrolytes: HClO_4_ (Sigma Aldrich, 70%, 99.999% trace metal basis), KOH (Supelco, 99.995% Suprapur), H_2_SO_4_ (Supelco, 96% Suprapur), and ultra-pure water (Milli-Q, resistivity ≥18.2 MΩ cm).

### *Operando* synchrotron RefleXAFS experiment

A hat-shaped polycrystalline platinum (99.999%) sample was manufactured and polished by Surface Preparation Laboratory, Netherlands. The grain structure and surface orientations were measured with electron back-scattering diffraction (EBSD) as shown in the SI, Fig. S1. The top of the sample has a diameter of 7.5 mm and the bottom of the sample 14 mm, and the height is 4 mm. Prior to the experiment the sample surface was cleaned by cycles of Argon sputtering and annealing in 1 × 10^−6^ mbar of O_2_ at 800C in a UHV system to ensure a clean and flat surface for the total reflection experiments. The last annealing step was done without oxygen. After introducing the sample to the electrochemical flow cell, the sample surface was conditioned by cycling the potential between 0 and 1.4 V 25 times at 100 mV s^−1^.

The *operando* RefleXAFS measurements were performed at the Balder beamline at MAX IV, Sweden. The sample contained in our polyether ether ketone (PEEK) electrochemical flow cell dedicated to *operando* synchrotron experiments (shown in Fig. 1(a) and described in ref. 56) was mounted on the RefleXAFS goniometer assembly, allowing for adjusting the height of the sample surface and the incidence angle relative to the X-ray beam. The intensity of the incident beam was measured using an ionization chamber upstream of the sample. Downstream of the sample, a slit system was used to define the acceptance angle of the reflected beam, which was measured using an ionization chamber tilted parallel to the reflected beam. For the experiments, an incidence angle of 0.2 degrees was used. RefleXAFS spectra were measured at the Pt L_3_ edge. To minimize beam-induced effects, the electrolyte was constantly flowing using a peristaltic pump, and the intensity of the X-ray beam was reduced. CVs illustrating the effect of the X-ray beam are shown in the SI, Fig. S2.

The PEEK electrochemical flow cell was fitted with polytetrafluoroethylene (PTFE) tubing. The electrolyte was contained in a perfluoroalkoxy (PFA) bottle, and a peristaltic pump was used to circulate the electrolyte. The synchrotron flow cell was cleaned by flowing a solution of 1 g l^−1^ K_2_MnO_4_ and 0.5 M H_2_SO_4_ through the cell overnight. The cell was then flushed through with a mixture of ∼0.5 M H_2_SO_4_ and ∼0.5 M H_2_O_2_ for 1 hour and afterwards flushed with several liters of ∼80 °C ultra-pure water. PFA bottles for the synchrotron experiment were cleaned by submerging in a solution of 1 g l^−1^ K_2_MnO_4_ and 0.5 M H_2_SO_4_ overnight. All parts were then rinsed in ultra-pure water and submerged in a mixture of ∼0.5 M H_2_SO_4_ and ∼0.5 M H_2_O_2_ for 10 minutes. Afterwards, all parts were rinsed and boiled 3 times in ultra-pure water.

Electrolytes were made using ultra-pure water and 70% HClO_4_ and were degassed with either 5N Ar or 5N O_2_. A platinum rod (MaTeck 99.999%) was used as a counter electrode and a mini hydrogen reference electrode from Gaskatel was used. A Bio-Logic VSP300 potentiostat was used for the electrochemical experiments at the synchrotron, and all potentials are reported *versus* the reversible hydrogen electrode (RHE) scale.

### Lab-based electrochemical experiments

The one compartment glass cell for RDE experiments was cleaned by submerging it in a solution of 1 g l^−1^ K_2_MnO_4_ and 0.5 M H_2_SO_4_ overnight. All parts were then rinsed in ultra-pure water and submerged in a mixture of ∼0.5 M H_2_SO_4_ and ∼0.5 M H_2_O_2_ for 10 minutes. Afterwards, all parts were rinsed and boiled 3 times in ultra-pure water.

A 5 mm diameter polycrystalline platinum disc was used as the working electrode and was mounted in a commercial RDE sample holder (Pine Instruments). The sample surface was prepared by polishing using diamond suspensions of 3 µm, 1 µm, and 0.25 µm (MetaDi polycrystalline, Buehler) for two minutes each. Afterwards, the sample was sonicated for 5 minutes in a mixture of ultra-pure water and ethanol, followed by sonication for 5 minutes in ultra-pure water. Once the sample was inserted into the cell, it was activated by 100 CV cycles between 0 and 1.4 V at 100 mV s^−1^ to establish a steady-state polycrystalline platinum voltammogram, as shown in the SI, Fig. S25.

Blank voltammograms (in Ar) were measured without rotation, and experiments in O_2_ were recorded using a rotation rate of 2500 rpm. Scan rates varied between 10 and 250 mV s^−1^. Before each voltammogram or LSV, three conditioning cycles between 0 and 1.4 V at 100 mV s^−1^ were performed to ensure reproducibility of the results and remove possible contaminations from the surface. Solution resistance was measured using electrochemical impedance spectroscopy (EIS) and determined from the Nyquist plot where the curve crosses the real axis. For 0.1 M HClO_4_ in the glass cell, the solution resistance was always around ∼20 ohm. *iR* compensation at 85% was used in the software.

Electrolytes were made using ultra-pure water and 70% HClO_4_ and were degassed with either 5N Ar or 5N O_2_. Experiments with KOH (a PFA plastic cell was used for alkaline experiments together with an RHE reference electrode (Gaskatel, HydroFlex)) and H_2_SO_4_ are shown in the SI, Fig. S14 and S15. A homemade RHE consisting of a platinum wire in a separate compartment bubbled with H_2_ was used as a reference electrode. A platinum wire was used as the counter electrode. A Bio-Logic VSP300 potentiostat was used for the electrochemical experiments, and all potentials are reported *versus* the RHE scale.

### *Ex situ* X-ray photoelectron spectroscopy

XPS was measured *ex situ* in vacuum using a Scientia lab-based system equipped with a monochromatic Al K-edge X-ray source described in ref. 57. The same polycrystalline platinum sample was used for the XPS and the RDE experiments, and the sample surface was prepared as described above. XPS was measured after taking out the sample under potential control, rinsing with ultra-pure water, and drying with compressed air. The sample was then immediately transferred into the XPS chamber. The low level of carbon detected in the survey spectrum (shown in the SI, Fig. S17) indicates a high level of cleanliness even after transferring from the electrochemical cell to the chamber. Further details of data analysis are given in the SI.

### Microkinetic modeling

To simulate our model, we used ElectroKitty a Python electrochemistry simulation package.^44^ After coding the Pt oxidation model, we used a Python CMA-ES package to optimize this model's parameters.^58,59^ The fits were made using five CVs at different scan rates and upper vertex potential to allow the model to capture a broad region of experimental conditions. The loss function employed was thus an evenly averaged relative root mean squared function evaluated for each CV separately. For the ORR, the formal potential was set at 1.05 V, with a rate constant of 2.5 m^3^ mol^−1^ s^−1^ and a charge transfer parameter of 0.5. The O_2_ concentration was set to 4.85 mM and the diffusion rate to 3.5 × 10^−9^ mol m^−2^. Further details can be found in the SI.

## Author contributions

Alfred Larsson (conceptualization, data curation, formal analysis, funding acquisition, investigation, software, writing – original draft, writing – review & editing), Andrea Grespi (investigation, writing – review & editing), Ozbej Vodeb (data curation, formal analysis, investigation, software, writing – review & editing), Karen van den Akker (investigation, writing – review & editing), Auden Ti (investigation, writing – review & editing), Claire Berschauer (investigation, writing – review & editing), Alexander Imre (investigation, writing – review & editing), Philip Miguel Kofoed (investigation, writing – review & editing), Estephania Lira (investigation, writing – review & editing), Mahesh Ramakrishnan (methodology, resources, writing – review & editing), Stuart Ansell (methodology, resources, writing – review & editing), Justus Just (methodology, resources, writing – review & editing), Henrik Grönbeck (data curation, formal analysis, investigation, software, writing – review & editing), Ulrike Diebold (supervision, funding acquisition, writing – review & editing), Edvin Lundgren (conceptualization, supervision, funding acquisition, writing – review & editing), Lindsay Merte (conceptualization, supervision, funding acquisition, writing – review & editing), Dusan Strmcnik (supervision, resources, writing – review & editing), Rik Mom (conceptualization, supervision, resources, writing – review & editing), and Marc Koper (conceptualization, supervision, resources, writing – review & editing).

## Conflicts of interest

There are no conflicts to declare.

## Supplementary Material

EY-004-D6EY00014B-s001

## Data Availability

Supplementary information is available. See DOI: https://doi.org/10.1039/d6ey00014b. Data can be made available upon reasonable request.
