# Supplementary material for: Platinum surface oxides govern the cathodic overpotential of the oxygen reduction reaction
Source: EES Catal. 2026 Mar 18;4(3):754–65. doi: 10.1039/d6ey00014b (PMC12997406; doi:10.1039/d6ey00014b)
Supplement: EY-004-D6EY00014B-s001 [file EY-004-D6EY00014B-s001.pdf]

Supplementary Information (SI) for EES Catalysis.
This journal is © The Royal Society of Chemistry 2026

Supporting information for:

Platinum Surface Oxides Govern the Cathodic Overpotential of the Oxygen Reduction Reaction

Alfred Larsson[1,2], Andrea Grespi[2], Ozbej Vodeb[1,3,4], Karen van den Akker[1], Auden Ti[2], Claire Berschauer[2,5], Alexander M. Imre[6], Philip Miguel Kofoed[7], Estephania Lira[2], Mahesh Ramakrishnan[8], Stuart Ansell[8], Justus Just[8], Henrik Grönbeck[9], Ulrike Diebold[6], Edvin Lundgren[2], Lindsay R. Merte[7], Dusan Strmcnik[3], Rik Mom[1], and Marc T.M. Koper[1]*

[1]*Leiden Institute of Chemistry, Leiden University, Leiden, The Netherlands*

[2]*Division of Synchrotron Radiation Research, Lund University, Lund, Sweden*

[3]*Department of Materials Chemistry, National Institute of Chemistry, Ljubljana, Slovenia*

[4]*Jozef Stefan International Postgraduate School, Jamova cesta 39, 1000 Ljubljana, Slovenia*

[5]*Wallenberg Material Science Initiative for Sustainability, Division of Synchrotron Radiation Research, Lund University, Lund, Sweden*

[6]*Institute of Applied Physics, TU Wien, Vienna, Austria*

[7]*Materials Science and Applied Mathematics, Malmö University, Malmö, Sweden.*

[8]*MAX IV Laboratory, Lund University, Lund, Sweden*

[9]*Department of Physics and Competence Centre for Catalysis, Chalmers University of Technology, Göteborg, Sweden*

**Corresponding authors: email: alfred.larsson@fysik.lu.se, m.koper@lic.leidenuniv.nl*

Table of Contents

Details on the synchrotron experiment 3
- Electron backscatter diffraction (EBSD) of the platinum polycrystal used for synchrotron experiments 3
- Effect of X-ray beam on CV 3
- Experimental setup for the synchrotron experiment 4
- RefleXAFS measurement 4
- RefleXAFS data treatment 5
- In situ RefleXAFS oxide signal during CV and LSV 9
- Data for adsorbing anion and alkaline media 10

Additional details on XPS 12
- Survey spectrum 12
- Justification of two additional doublets in Pt 4f upon oxidation 12
- Calculations of core-level shifts using DFT 13
- Fitting of Pt 4f spectra 14
- Platinum oxide thickness calculations 17
- Presence of Pt-O bonds from O 1s 17

Supporting electrochemical data 18

Microkinetic modeling 21
- Supporting figures 22

References 24

Details on the synchrotron experiment

Electron backscatter diffraction (EBSD) of the platinum polycrystal used for synchrotron experiments

Figure S1 shows the grain orientation and microstructure of the platinum polycrystal used for the synchrotron experiment. Note, however, that upon oxidation-reduction cycles used for activation of the sample surface, the real surface structure no longer represents the bulk truncation of the grain.

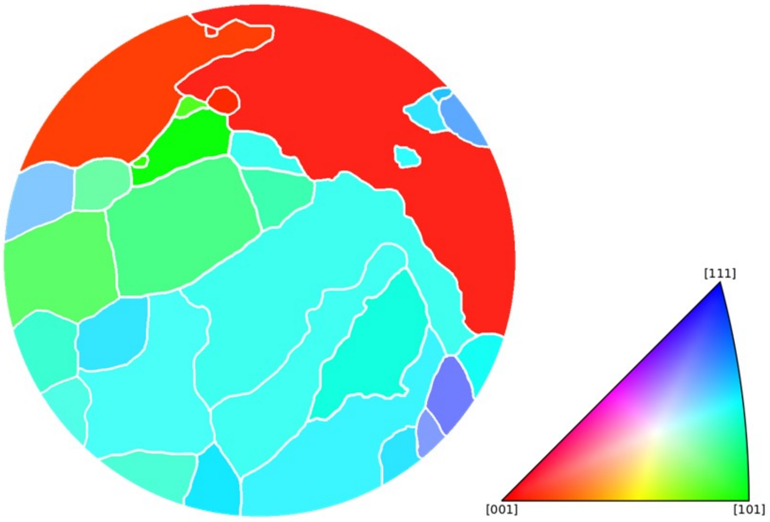

Figure S1: BESD map of the grain orientation for the polycrystalline platinum sample used for the synchrotron experiment. The diameter of the sample surface is 7.5 mm.

Effect of X-ray beam on CV

Figure S2 shows blank CVs (Ar saturated 0.1 M $HClO_4$) in the synchrotron flow cell measured with the X-ray beam on and off. A small increase in the oxidation current can be seen, similar to what has been observed for Pt(111) [1].

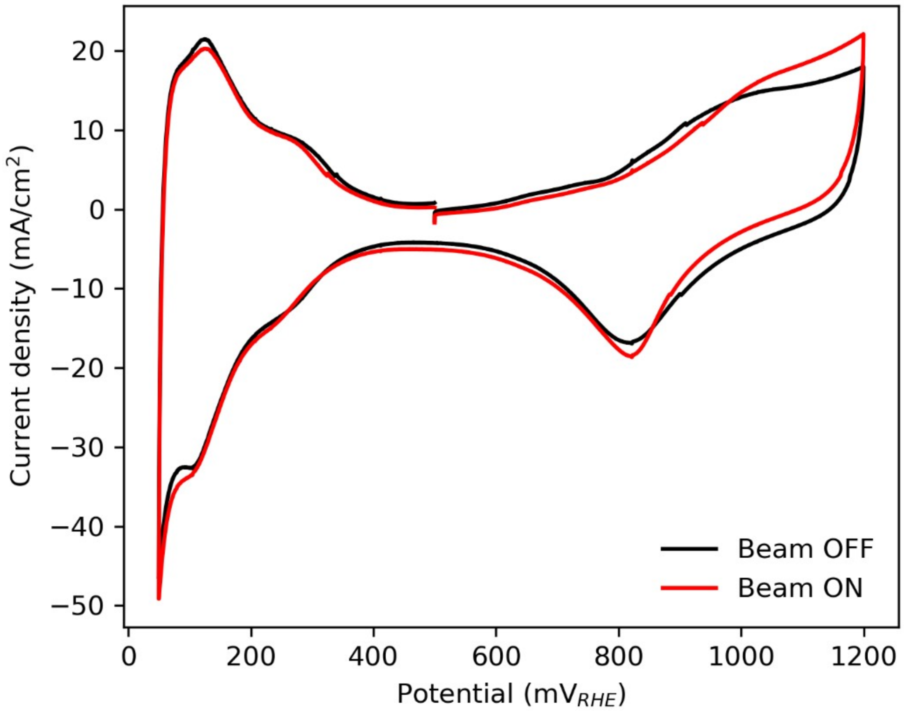

Figure S2: CV of polycrystalline platinum in Ar-saturated 0.1 M $HClO_4$ measured in the synchrotron flow cell at the beamline with X-ray beam on or off.

Experimental setup for the synchrotron experiment

Figure S3 shows pictures of the experimental setup for the synchrotron experiment at the BALDER beamline at MAX IV, Sweden.

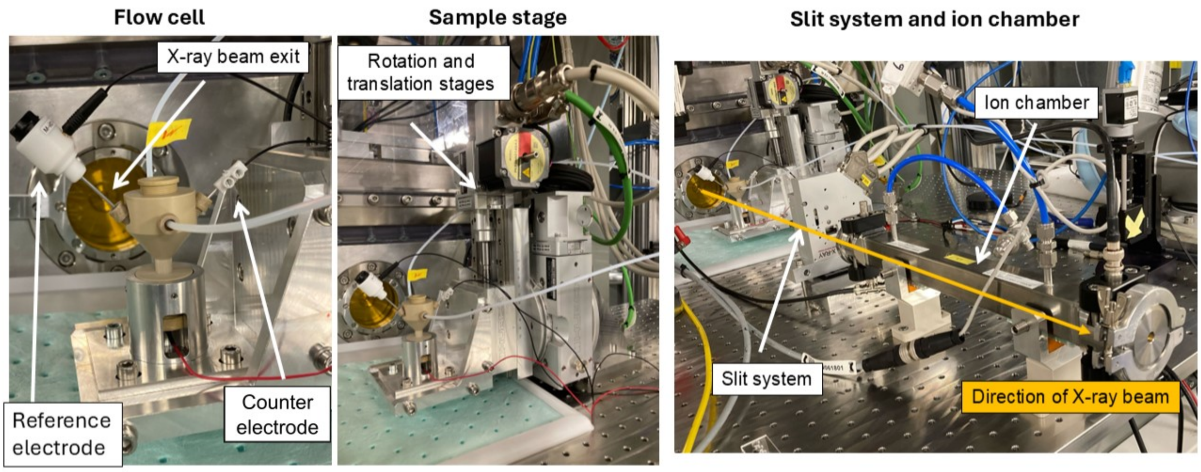

Figure S3: Pictures of the experimental setup used for the synchrotron experiment.

RefleXAFS measurement

The energy-dependent reflectivity was measured at a fixed incidence angle of 0.2 degrees, well below the critical angle of Pt (0.354 degrees at 11560 keV)[2]. The acceptance of the reflected beam was determined by the detection slits placed in front of the ion chamber. A 1 mm vertical slit opening was

used, allowing only the reflected X-rays to be detected while blocking the direct beam and stray scattering. A scan of the vertical slit position across the reflected beam while the sample is in the reflection condition (0.2 degrees incidence angle) with a slit opening of 0.1 mm is shown in Figure S4. The position of the slits with a 1 mm opening is marked in red to illustrate the part of the reflected beam caught by the slits. The intensity of the measured reflectivity was around 50% of the direct beam.

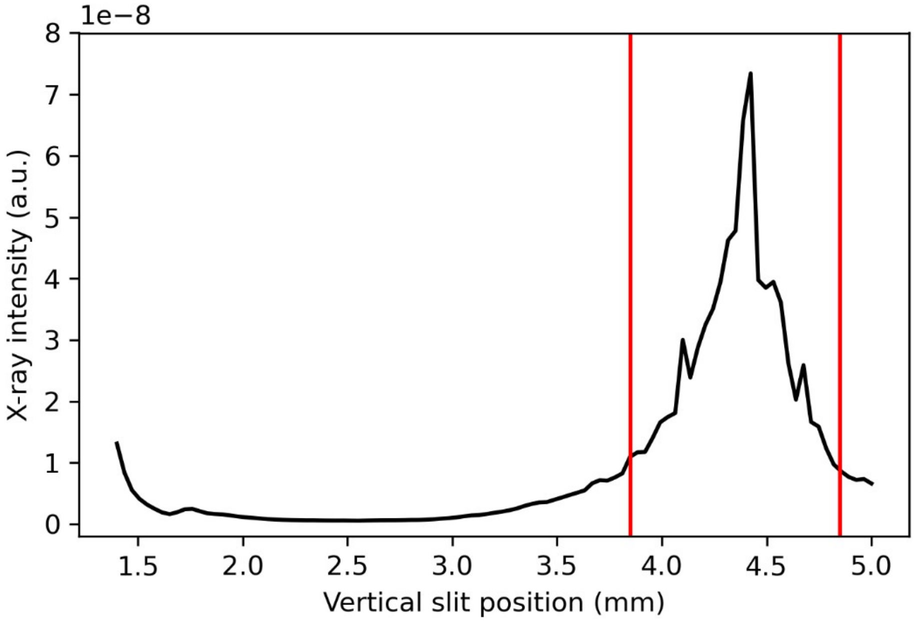

Figure S4: Scan of the vertical detection slit across the reflected X-ray beam with a slit opening of 0.1 mm. For the measurement, a alit opening of 1 mm was used to catch the majority of the reflected beam as indicated in red.

RefleXAFS data treatment

The measured RefleXAFS data were normalized using LARCH[3] in Python as shown in Figure S5. All spectra are treated as the logarithm of the measured energy dependent reflected intensity I_R divided by the intensity of the incoming beam, I_0. Note that due to the nature of the reflection process and especially the anomaly in the refractive index produced by absorption, total-reflection spectra may differ from those obtained in transmission mode. Signals may also result from a superposition of contributions from the evanescent wave absorption during the reflection process and transmission through island-like features under shallow incidence and reflection angle. Assuming a shallow incidence angle (far below the critical angle) the spectral shape of the absorption fine structure comes close to traditional transmission data[4]. Significant deviations of the reflection spectra compared to conventional transmission data are not evident in our data, which appear to be well explained in terms of absorption in the near-surface of the sample. Thus, for easy visualization and comparison with the reference X-ray absorption near-edge spectra (XANES), we normalize the energy-dependent reflectance in the same way as a traditional XANES data treatment, i.e. normalizing the $-ln(I_R/I_0)$.

Examples of representative high-quality spectra used for comparison to references and fast acquisition spectra used for time resolved studies are shown in Figure S6. Spectra with an acquisition time of 3 minutes were smoothed using a Savitzky–Golay filter with a window length of 5 and a polynomial order of 1. Spectra with an acquisition time of 1 second were

smoothed using a Savitzky–Golay filter with a window length of 15 and a polynomial order of 1. Smoothing of the data has no effect on the shape of the difference spectrum, as shown in Figure S7.

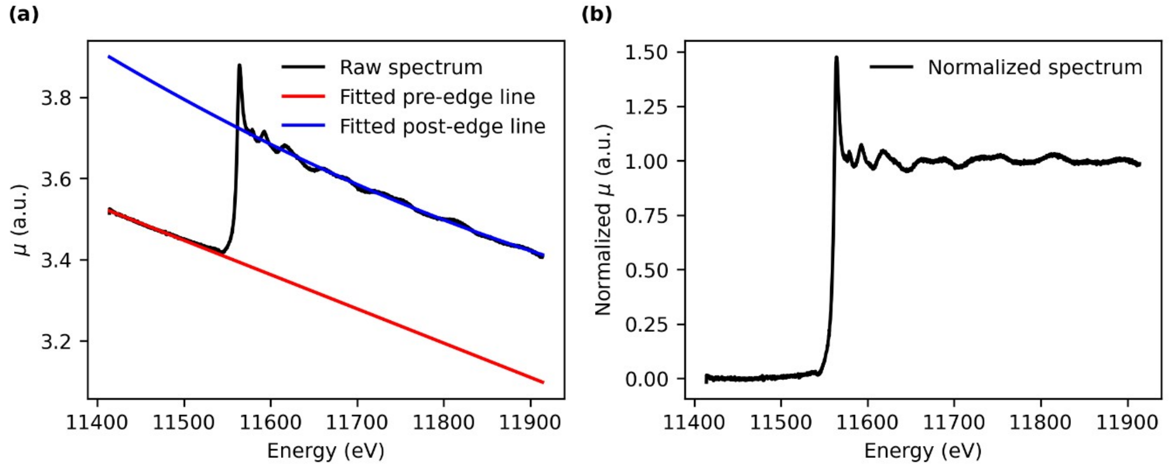

Figure S5: (a) Raw spectrum together with fitted pre- and post-edge lines. (b) Normalized spectrum.

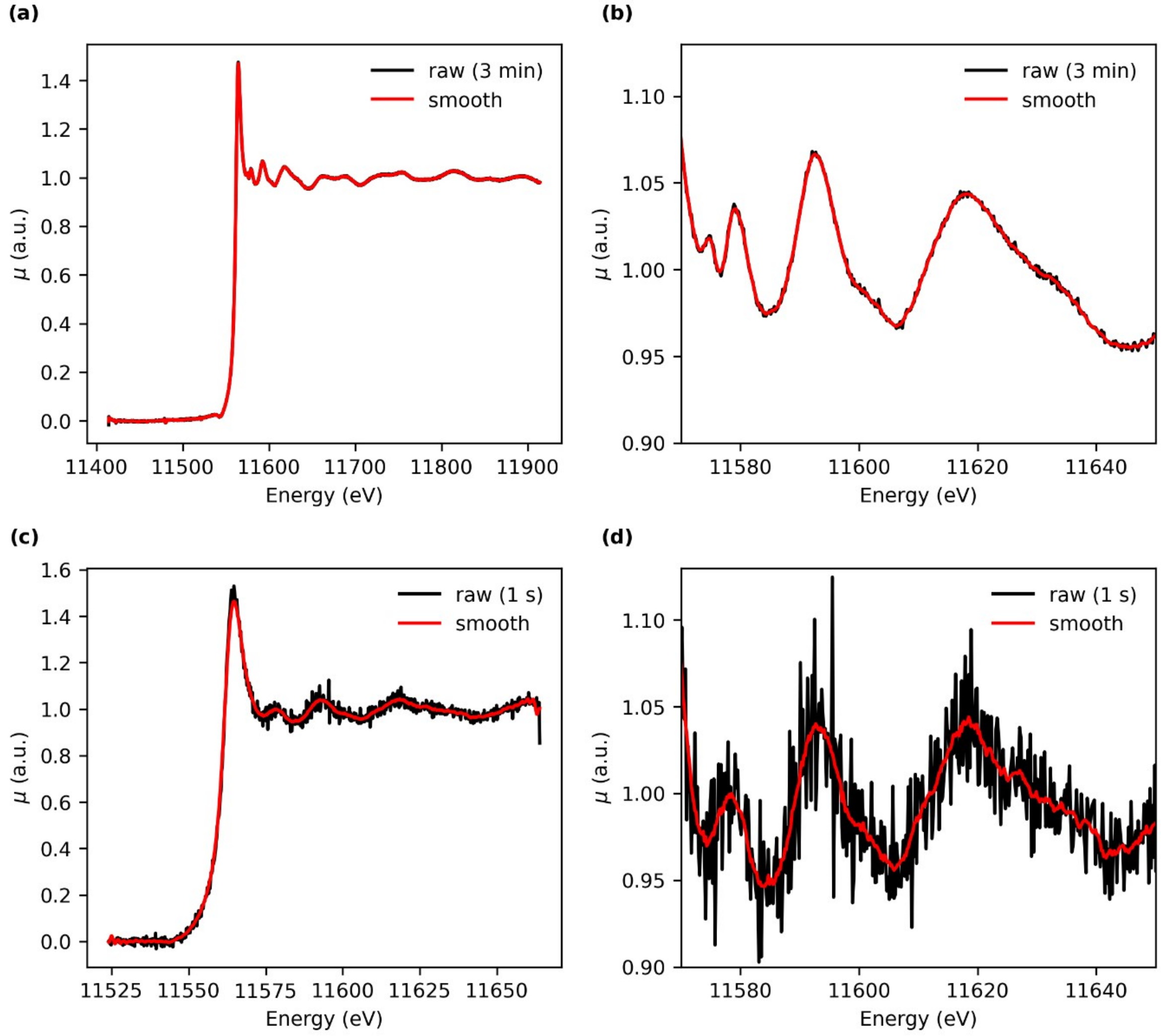

Figure S6: (a) High-quality spectrum with an acquisition time of 3 minutes and smoothed spectrum. (b) magnified view of (a). (c) High time resolution spectrum with an acquisition time of 1 second and smoothed spectrum. (d) magnified view of (c).

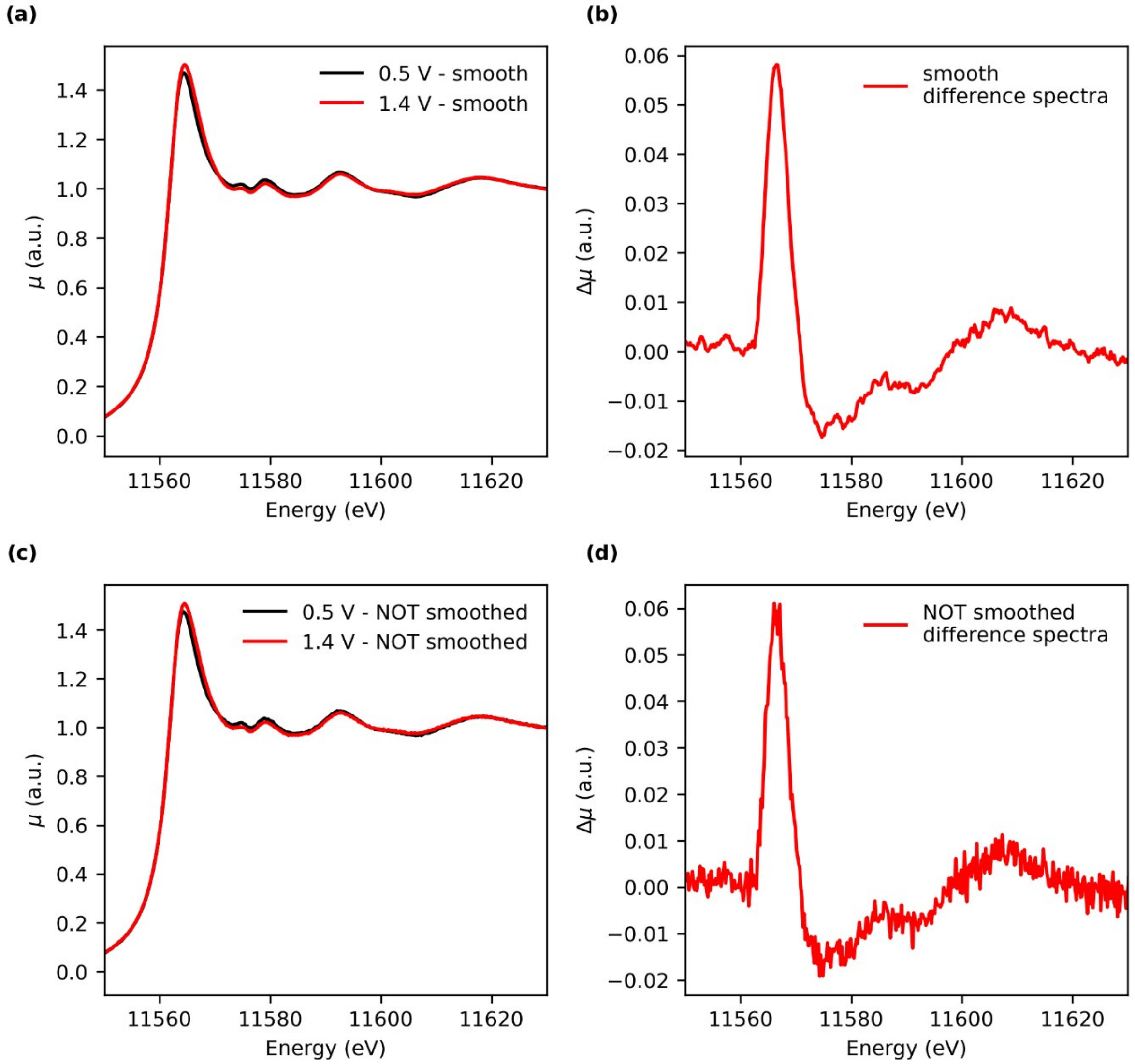

Figure S7: (a) Smoothed RefleXAFS spectra at 0.5 and 1.4 V. (b) Difference spectra calculated from smoothed spectra in (a). (c) RefleXAFS spectra at 0.5 and 1.4 V. (d) Difference spectra calculated from smoothed spectra in (c). Note that smoothing does not have any effect on the spectral shape.

RefleXAFS at fixed potentials

Figure S8 shows examples of RefleXAFS spectra measured at different upper vertex potentials compared to a spectrum measured at 500 mV.

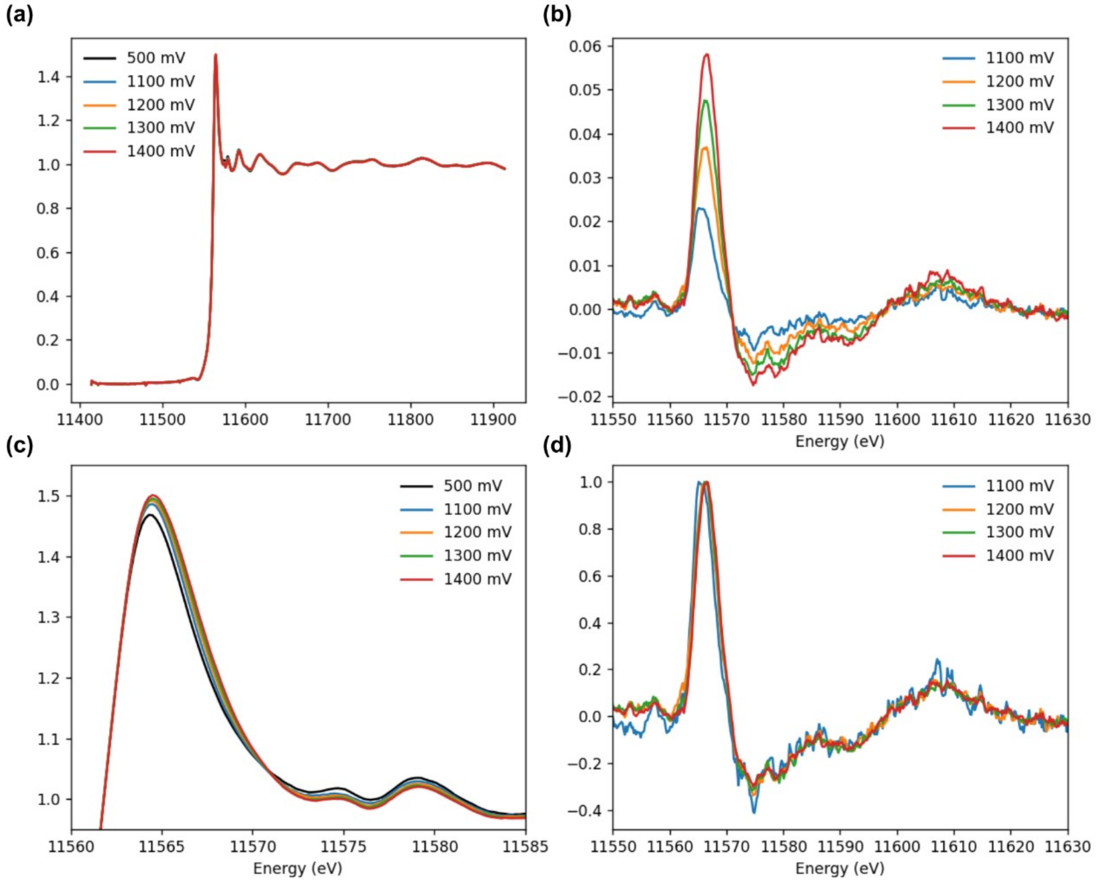

Figure S8: (a) Full RefleXAFS spectra as a function of potential. (b) Difference spectra where the spectrum at 500 mV was subtracted. (c) Magnified view of (a). (d) Scaled difference spectra to shows that the spectral shape of the oxide signal does not change with potential.

Extraction of oxide signal

Time-resolved RefleXAFS was performed with an acquisition time of 1 second while the potential was cycled. To extract the oxide signal, difference spectra were calculated where an averaged spectrum at reducing conditions was subtracted from every subsequent spectrum. The difference spectrum as a function of time during 3 CV cycles is shown in Figure S9. To extract the oxide signal from the difference spectra, the absolute in the region of 1163 eV to 1183 eV, (showing the white line and fine structure) was integrated. The oxide signal and electrochemical current as a function of time for 3 CV cycles are shown in Figure S9 (b). The average of 3 cycles is shown in red. The oxide signal for the 3 cycles as a function of potential is shown in Figure S10 (b), and the corresponding CV is shown in Figure S10 (a). Note the excellent overlap between the 3 cycles.

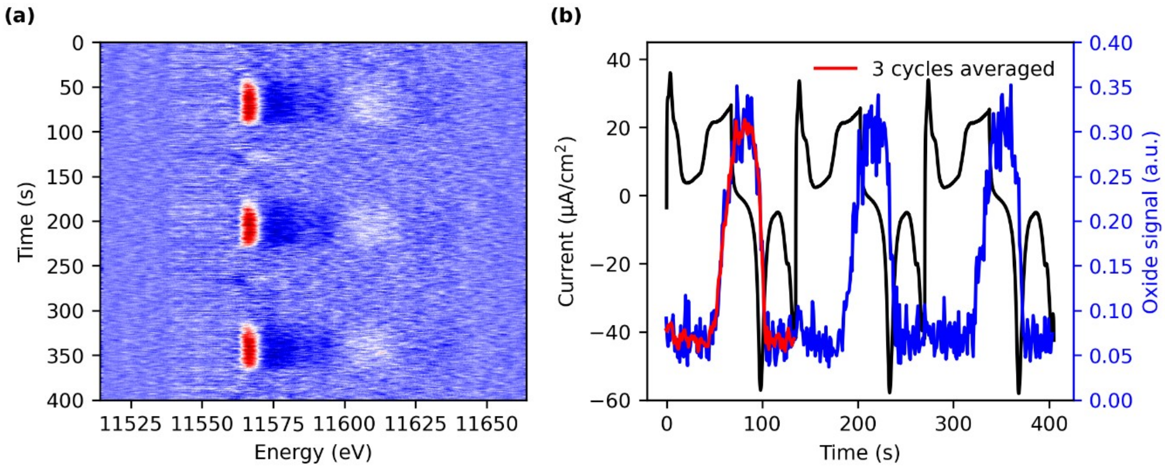

Figure S9: (a) Time resolved RefleXAFS difference spectra during CV with a time resolution of 1 second. (b) Integrated oxide signal (blue) as a function of time together with the electrochemical current (black). The average oxide signal of 3 CV cycles (red).

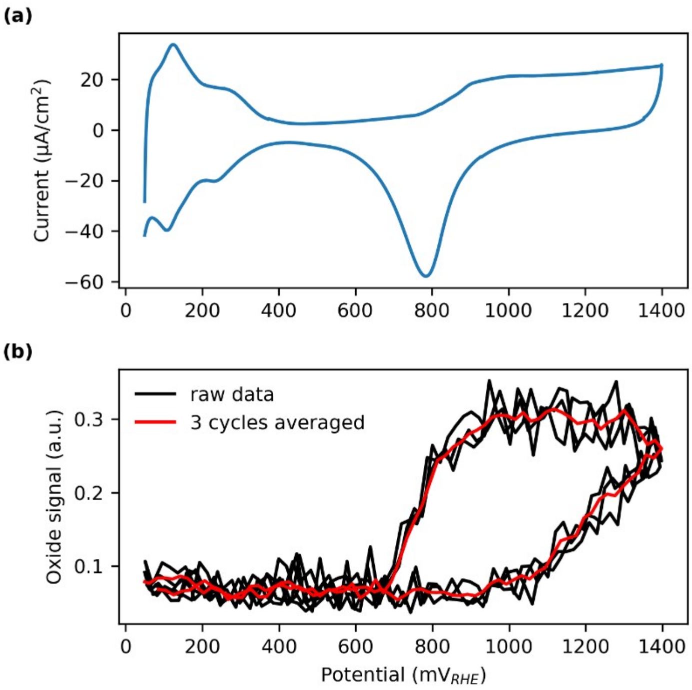

Figure S10: (a) CV between 0.05 and 1.4 V in 0.1 M $HClO_4$ (Ar saturated) measured at 20 mV/s in the synchrotron flow cell. (b) Oxide signal measured during CV in (a).

In situ RefleXAFS oxide signal during CV and LSV

Blank CVs at increasing upper vertex potential measured in the synchrotron flow cell are shown in Figure S11 (a). The corresponding oxide signals are shown in Figure S11 (b). LSVs starting at different upper vertex potentials after 30 s potential holds in O_2 saturated conditions are shown in Figure S12 (a), and the corresponding oxide signal is shown in (b).

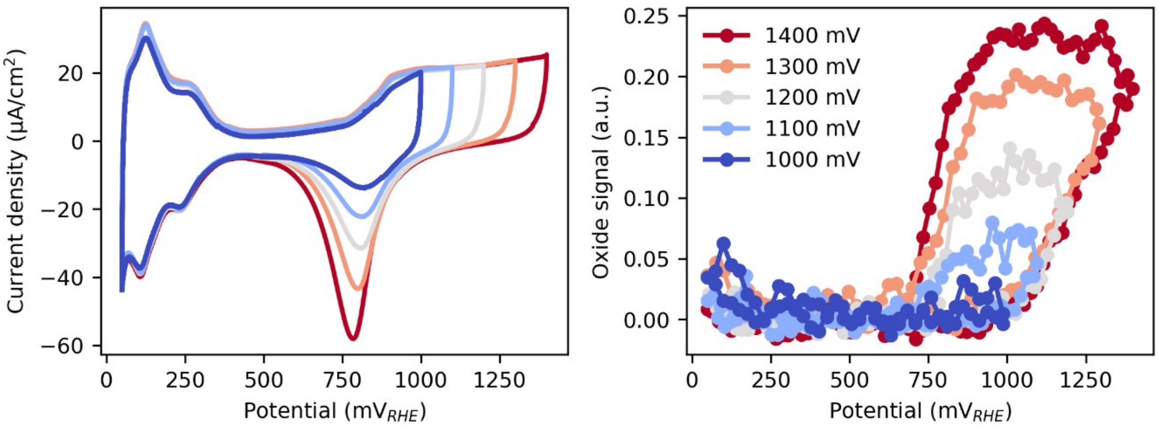

Figure S11: (a) blank CVs for varying upper vertex potential in the synchrotron flow cell (0.1 M $HClO_4$, Ar saturated, 20 mV/s) and the corresponding oxide signal in (b).

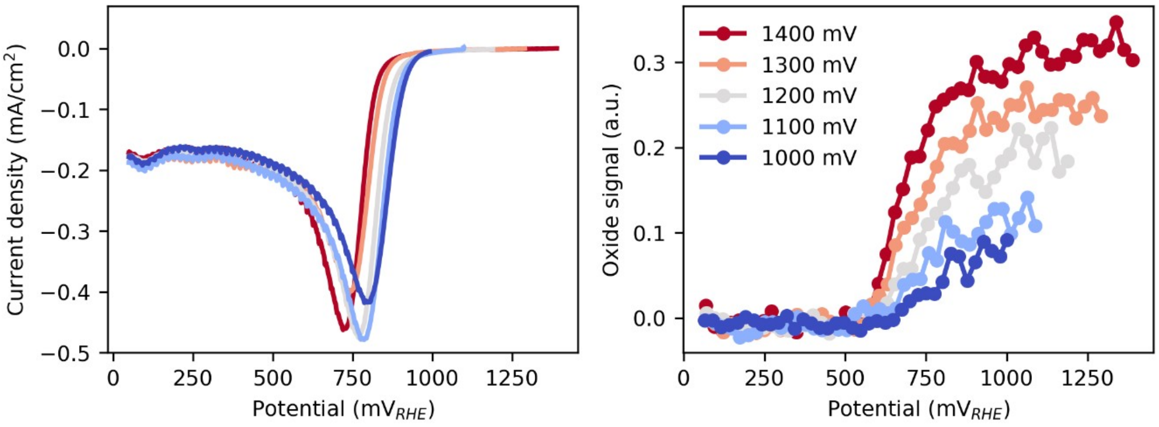

Figure S12: (a) ORR LSVs starting at varying upper vertex potentials measured in the synchrotron flow cell (0.1 M $HClO_4$, O_2 saturated, 20 mV/s) and the corresponding oxide signal in (b).

The difference in the oxide signal between the CV that ends at 1000 mV and the LSV that starts at 1000 mV after a 30 s potential hold at 1000 mV is caused by the potential hold time prior to the LSV. This is confirmed in Figure S13 (a), which shows the oxide signal vs time during a potential jump from 500 mV to 1000 mV. The oxide signal rapidly reaches the same value of around 0.1 as in Figure S12 at the beginning of the LSV. The difference Reflexafs spectrum is shown in Figure S13 (b), which confirms the presence of a surface oxide after a potential hold at 1000 mV. This proves that the difference in the oxide signal at the beginning of the LSV and in the CV in Figures S11 and S12 is not caused by any damage to the surface by going to 1.4 V but simply due to the development of the oxide during the 30-second potential hold prior to the LSV. This further proves that the difference in ORR kinetics for different initial oxide coverages is caused by the inhibition of the reaction by the surface oxides and not by damage to the surface by going to high anodic potentials.

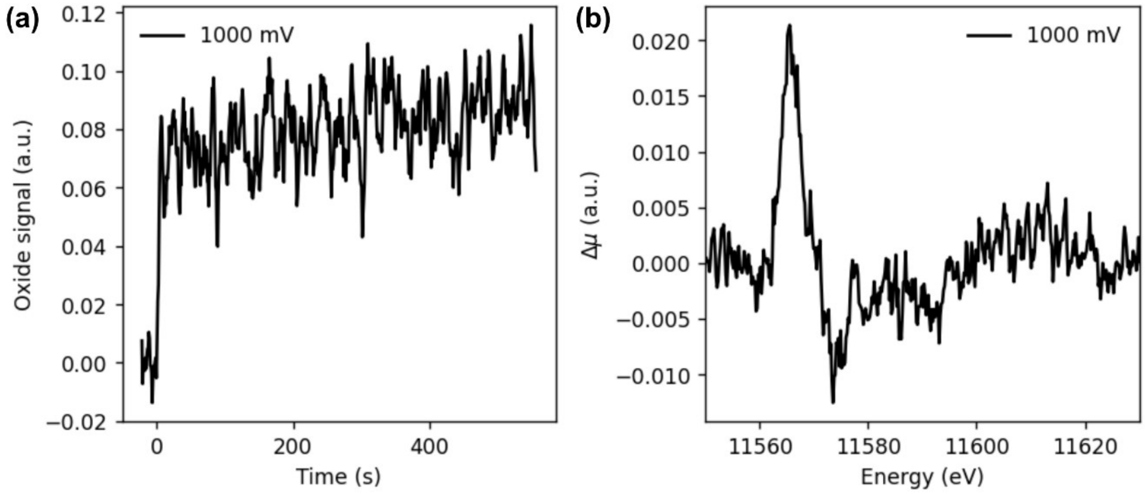

Figure S13: (a) Oxide signal as a function of time during a potential jump from 500 mV to 1000 mV. (b) Difference RefleXAFS spectrum after potential hold at 1000 mV

Data for adsorbing anion and alkaline media

Figure S14 shows CVs and cathodic LSVs in Ar and O_2-saturated solutions of KOH (alkaline media) and H_2SO_4 (adsorbing anion). The same behavior of oxide coverage modulated onset of ORR can be observed, as shown in Figure S15.

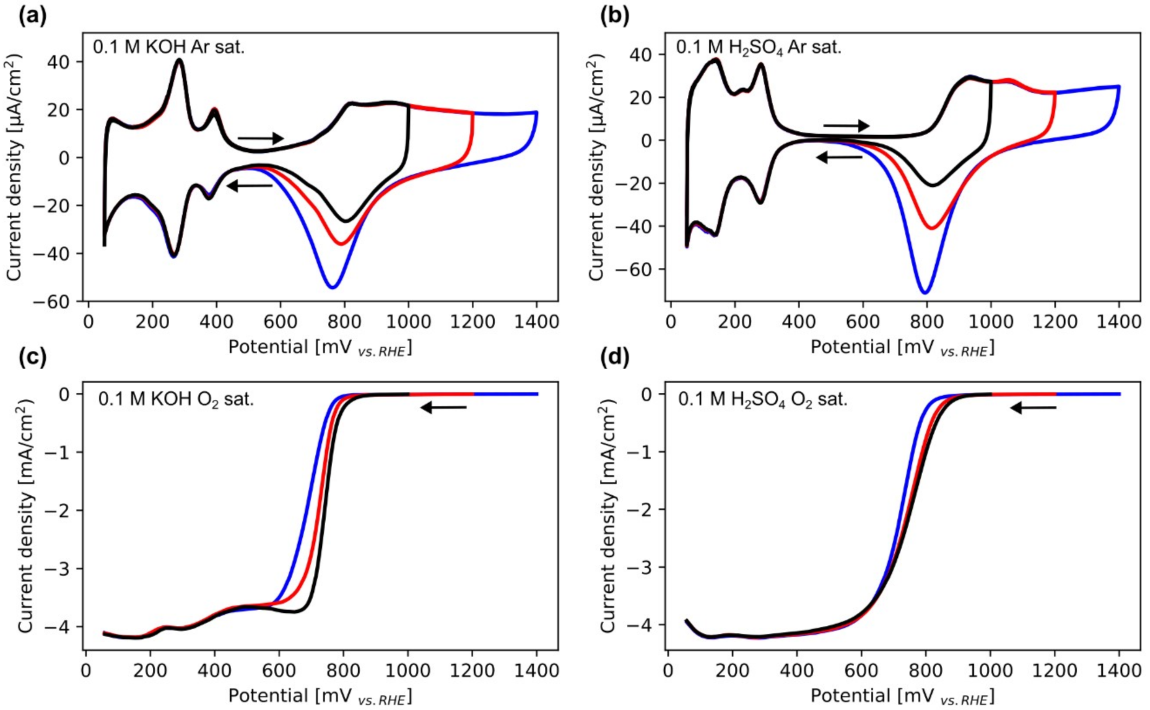

Figure S14: (a) CVs at 35 mV/s in Ar-saturated 0.1 M KOH. (b) CVs at 35 mV/s in Ar-saturated 0.1 M H_2SO4. (c) LSVs at 35 mV/s, 1600 rpm in O_2-saturated 0.1 M KOH. (d) LSVs at 35 mV/s, 1600 rpm in O_2-saturated 0.1 M H_2SO4.

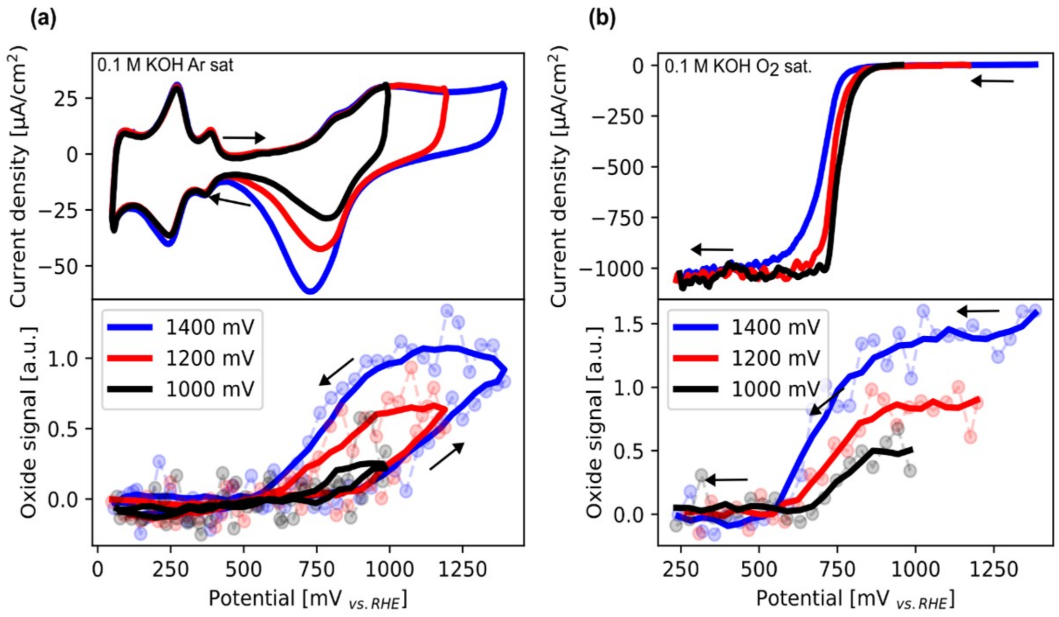

Figure S15: (a) CVs measured at 35 mV/s in Ar-saturated 0.1 M KOH using the synchrotron flow cell and corresponding RefleXAFS oxide signal. (b) Cathodic LSVs measured at 35 mV/s in O_2-saturated 0.1 M KOH using the synchrotron flow cell and corresponding RefleXAFS oxide signal

Absence of the effect of O_2 on oxidation kinetics

The effect of O_2 in the electrolyte on the oxidation kinetics was studied using chronoamperometry. Figure S16 shows the oxide signal as a function of time during a potential-step experiment from 0.5 V to 1.2 V in Ar and O2-saturated solutions. As shown, the presence of O_2 in the electrolyte does not affect the oxidation behavior. This goes in line with previous studies [5, 6].

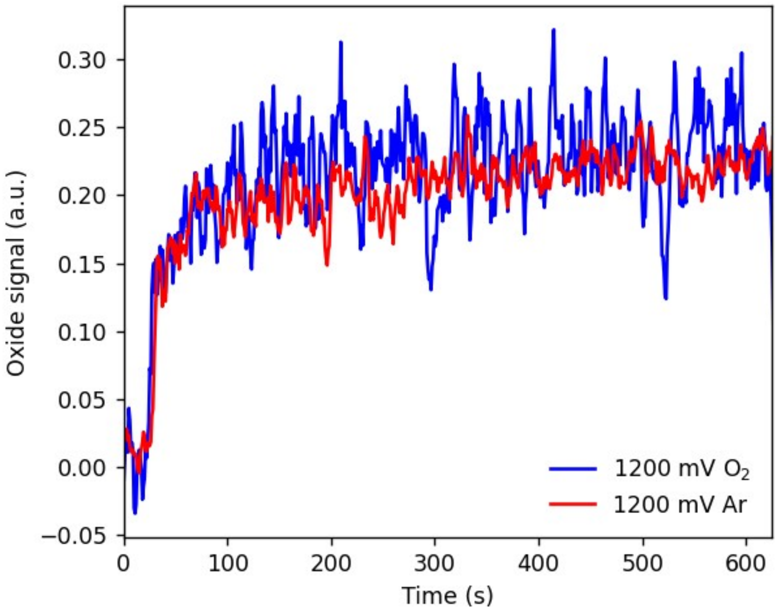

Figure S16: RefleXAFS oxide signal in Ar and O_2 saturation during a potential step experiment from 0.5 V to 1.2 V, illustrating that O_2 in the electrolyte does not affect the oxidation kinetics.

Additional details on XPS

Survey spectrum

Figure S17 shows a representative survey spectrum. Only Pt, O, and small amounts of C can be seen in the survey, indicating the cleanliness of the system.

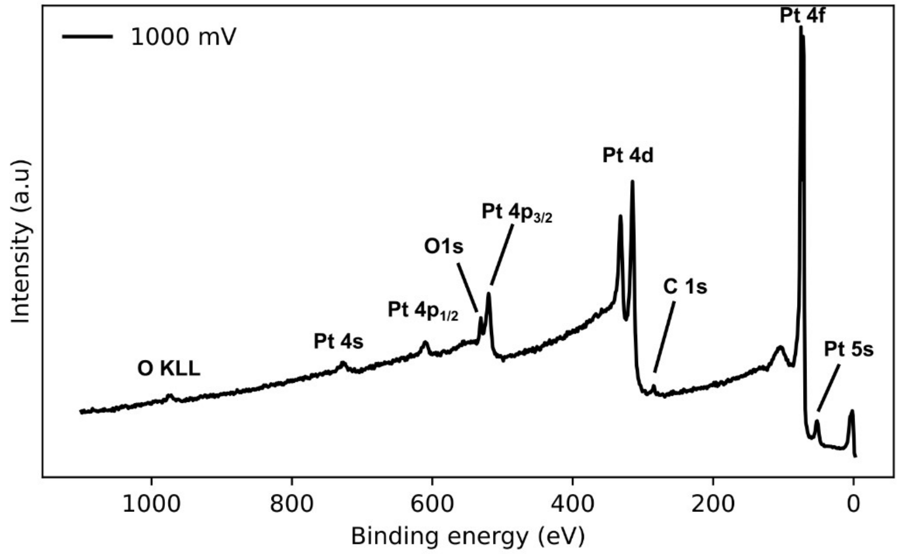

Figure S17: Survey spectrum measured after oxidation at 1 V for 10 minutes in 100 mM $HClO_4$.

Justification of two additional doublets in Pt 4f upon oxidation

Normalized spectra of the Pt 4f core level measured after taking the sample out under potential control at 0.5 V (reducing), 1 V, and 1.2 V are shown in Figure S18. As can be seen, new components are showing up after holding the sample at oxidizing potentials. To further clarify the changes in the spectra after oxidation, the spectrum at reducing conditions was subtracted to generate the difference spectra shown in Figure S19. Four peaks can be seen in the difference spectrum corresponding to two doublets. These doublets agree well with the binding energies of the 4-fold and 6-fold oxide structures, as shown by the vertical lines.

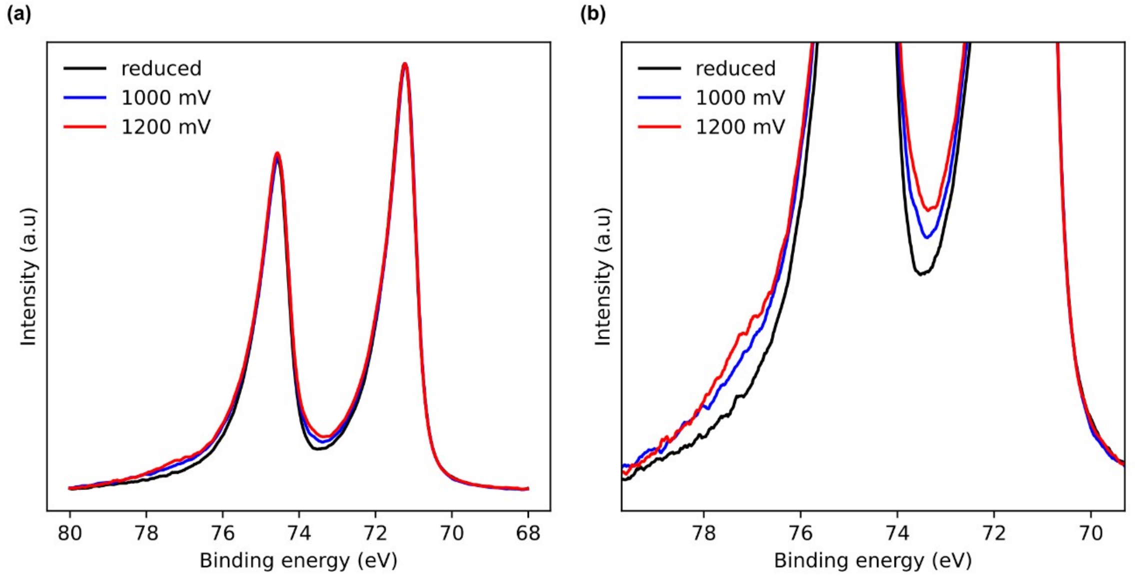

Figure S18: Background subtracted Pt 4f core levels normalized to the intensity of the Pt $4f_{7/2}$ peak. (a) full and (b) magnified view.

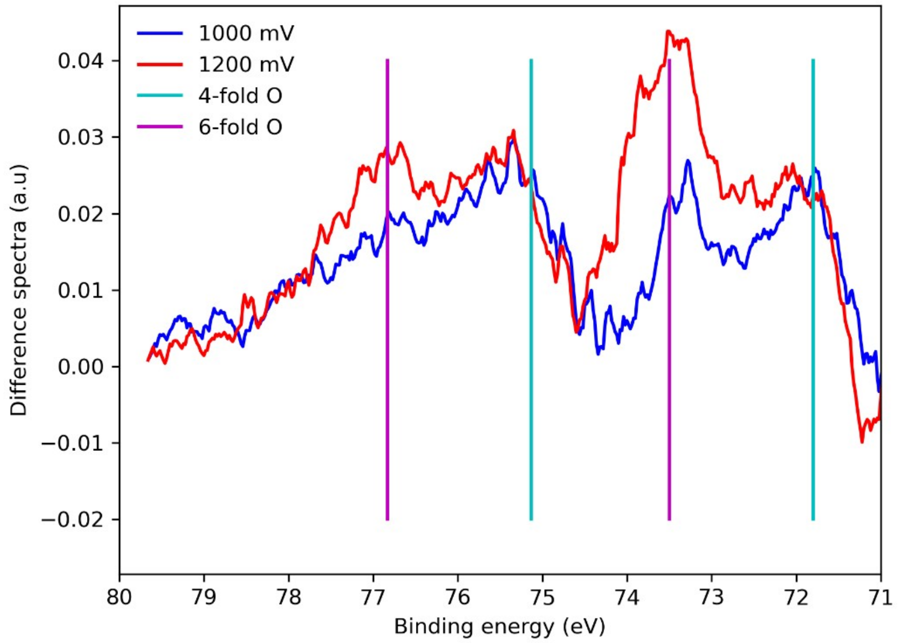

Figure S19: Difference spectra where the spectrum at reducing conditions was subtracted. Note the appearance of two doublets. The binding energies of the 4-fold and 6-fold oxide structures are indicated as vertical lines.

Calculations of core-level shifts using DFT

Density functional theory calculations were performed to facilitate the interpretation of the measured core level shifts. The calculations were performed using the Vienna Ab initio Simulation Package [7-10]. The Kohn–Sham orbitals were expanded in a plane wave basis, which was truncated at an energy of 480 eV. The exchange-correlation energy was calculated

using the Perdew, Burke, Ernzerhof (PBE) approximation [11]. The interaction between the valence electrons and the core was described with projected augmented wave potentials [12, 13]. Oxygen and platinum were treated with six and ten valence electrons, respectively. Integration over the Brillouin zone is approximated by finite sampling using a Γ−centered (6,6,1) grid for p(4x4) Pt(111) and a (6,1,1) grid for the (2x12) structure of oxidized Pt(110). A Gaussian smearing of the Fermi discontinuity was applied with a width of 0.1 eV. The (111) facet was investigated with surface slabs having 5 layers, whereas the Pt(110) surface was modelled with 8 layers. The bottom two layers were fixed to their bulk positions during geometry optimization. The slab models were separated by at least 12 Å of vacuum. The local geometry optimization was performed by the conjugate gradient method and the structures were considered to be optimized when all forces were smaller than 0.02 eV/Å. The surface core-level shifts (CLS) including final state effects were evaluated by use of a core potential with an electron hole in the 4f shell of Pt [14]. The CLSs were calculated with respect to a bulk reference of the bare Pt systems. The calculated core level shifts are shown in Figure S20. The calculated and used binding energies are shown in Table S1.

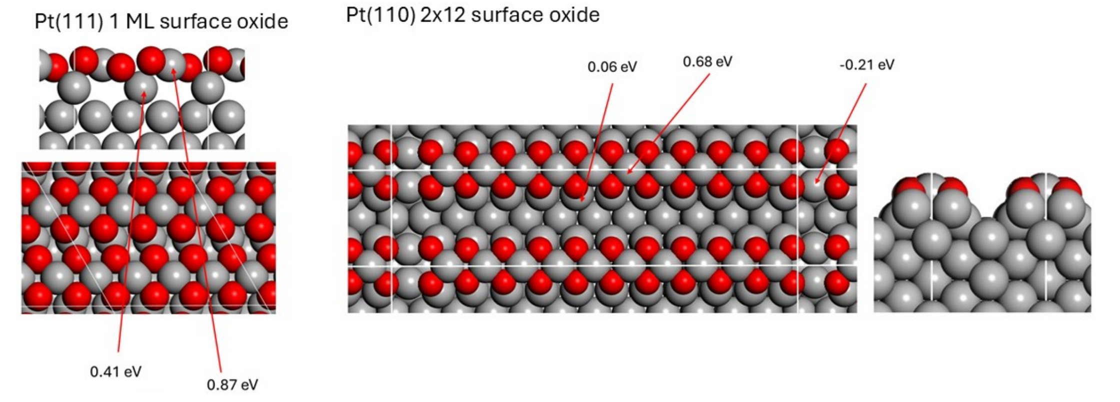

Figure S20: Core level shifts of platinum atoms in 4-fold oxide structures.

Table S1: XPS binding energies

Pt $4f_{7/2}$ Component	Binding energy (eV)	Core level shift (eV)
Metal	71	0
4-fold surface oxide, **experimental**	71.8	0.8
4-fold – **DFT**, Pt(110) 2x12 surface oxide	-	0.68
4-fold – **DFT**, Pt(111) 1 ML surface oxide	-	0.87
6-fold oxide, **Experimental**	73.47	2.47

Fitting of Pt 4f spectra

Peak fitting was performed using Python and the LMFIT package [15]. An asymmetric Voigt line shape [16] was used to fit the metal component of the Pt 4f core level, which displays asymmetry towards higher binding energy. All other peaks were fitted using Voigt line profiles. All spectra were background-subtracted using a Shirley background[17].

The Pt 4f spectrum at reducing conditions was fitted only with an asymmetric metallic doublet, as shown in Figure S21. All fitting parameters (width, asymmetry, binding energy) except the intensity were then locked before introducing additional oxide components.

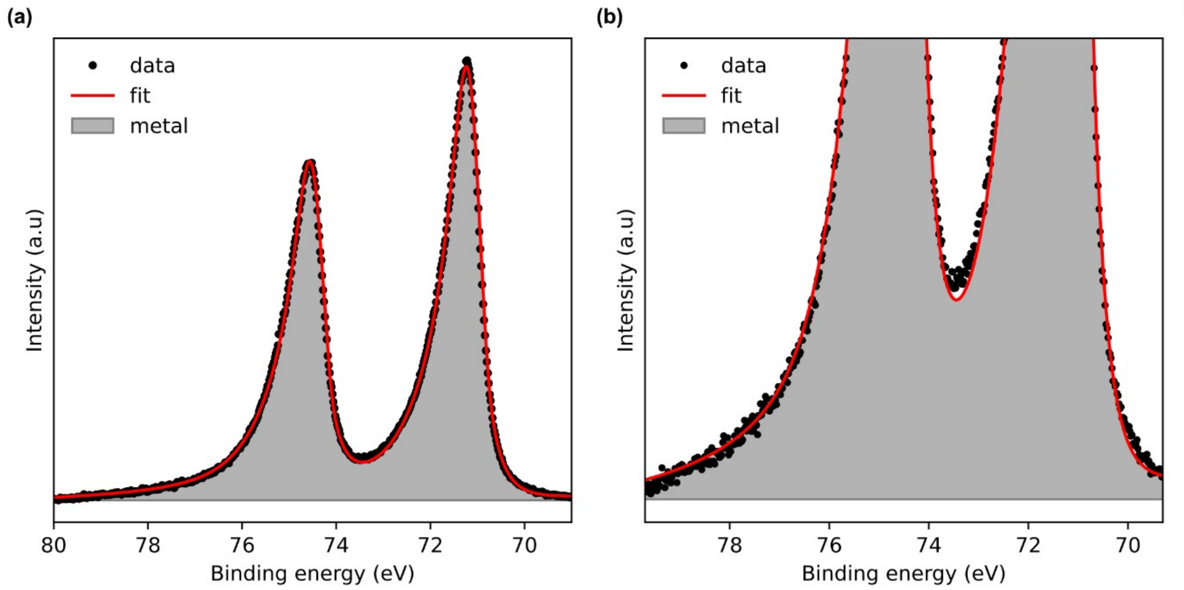

Figure S21: Full (a) and magnified (b) view of the fitted Pt 4f core level measured after taking the sample out at 0.5 V. Only an asymmetric metallic doublet is needed to fit the data.

To further demonstrate the need for two additional doublets (4-fold O and 6-fold O), the Pt 4f spectrum after polarization at 1 V (oxidizing conditions) was fitted first by only adding the 4-fold O component, as shown in Figure S22 (a) and (b). Then the spectrum was fitted by only adding the 6-fold O component, as shown in Figure S22 (c) and (d). Lastly, the spectrum was fitted with both the 4-fold and 6-fold components, as shown in Figure S22 (e) and (f), which yielded a lower chi-square and hence a better agreement with the experimental data.

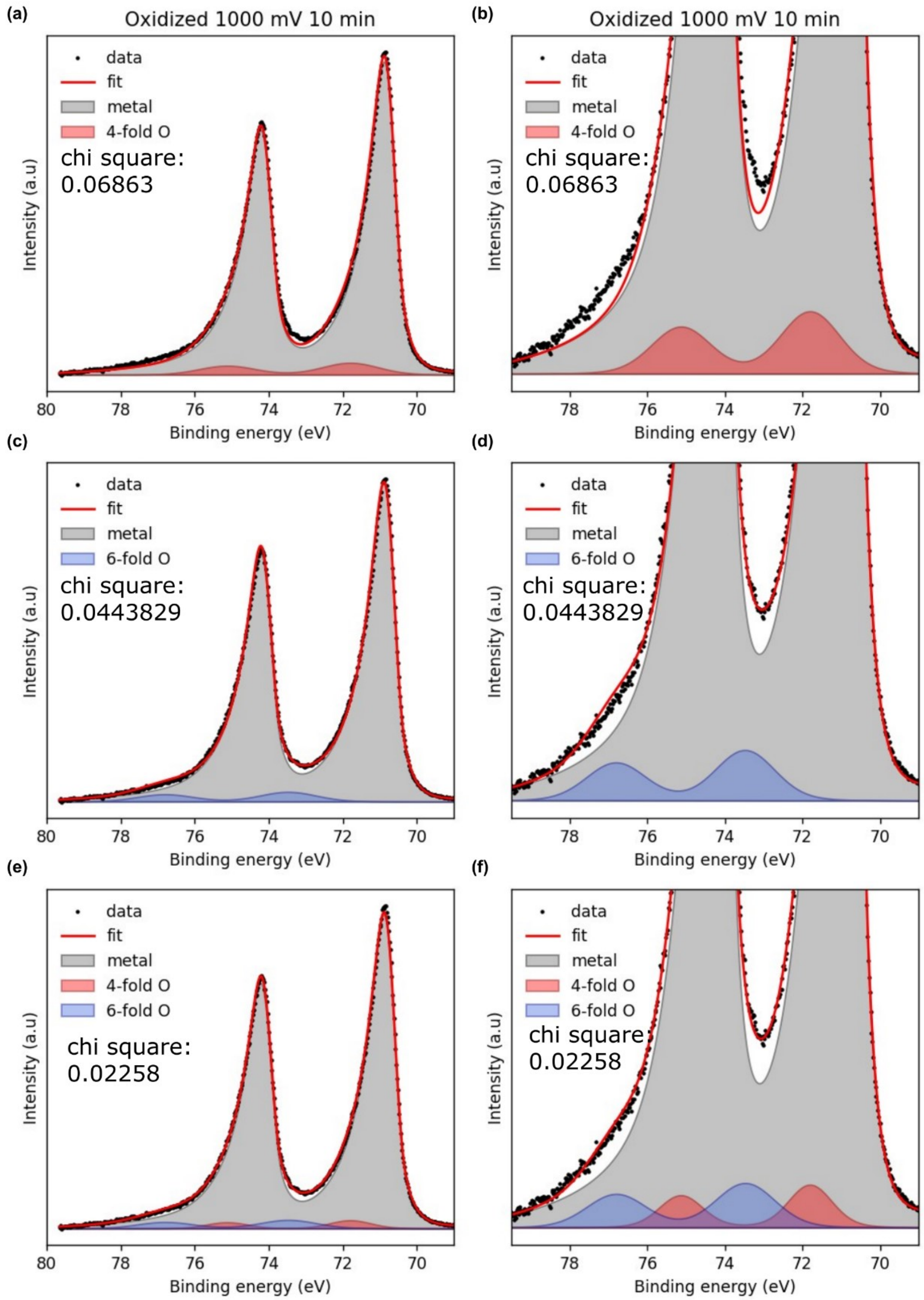

Figure S22: Fitting of Pt 4f XPS spectra. (a-b) full and magnified view fitted only with metallic and 4-fold component. (c-d) full and magnified view fitted only with metallic and 6-fold component. (e-f) full and magnified view fitted with metallic, 4-fold, and 6-fold components, which exhibit the lowest chi square.

Platinum oxide thickness calculations

The platinum oxide thickness was calculated with the well-established equation shown below.

$$d = \lambda_{oxide} \cdot \ln\left(\frac{1 + N_{metal}\lambda_{metal}I_{oxide}}{1 + N_{oxide}\lambda_{oxide}I_{metal}}\right)$$

where d is the oxide thickness, λ_x is the mean free path of the emitted photoelectrons in the given material. N_x is the atomic density in the given material, and I_x is the XPS intensity for component X. Here X is either the metal or the oxide. For the inelastic mean free path and atomic densities of the oxide, the values for PtO_2 are used since they are more well-defined. For the intensity of the oxide, the sum of the 4-and 6-fold components was used. All parameters used are listed in Table S2. The inelastic mean free path was calculated using the IMFP TPP2M software[18] and atomic densities were calculated using standard molar masses and density values. The calculated thickness is 2.5 Å at 1 V and 2.9 Å at 1.2 V after 10-minute potential holds.

Table S2: values used for the oxide thickness calculation based on XPS intensities

λ_{oxide} (Å)	λ_{metal} (Å)	N_{oxide} (mol/cm^3)	N_{metal} (mol/cm^3)
28.55	15.36	0.045	0.11

Presence of Pt-O bonds from O 1s

The presence of oxidic Pt-O bonds is also evidenced by looking at the O 1s core level. Figure S23 (a) shows the O 1s and Pt $4p_{3/2}$ from the survey spectra. As can be seen, when the potential is increased, the intensity of the O 1s signal increases relative to the Pt $4p_{3/2}$. Note that these are raw spectra. Figure S23 (b) shows higher resolution spectra of the O 1s core level, illustrating an increase of the 530 eV peak, characteristic of metal oxide bonds, as the potential is increased. This highlights the fact that most of the increase in the O 1s signal, and hence the O atoms present at the surface, is due to the formation of metal-oxide bonds.

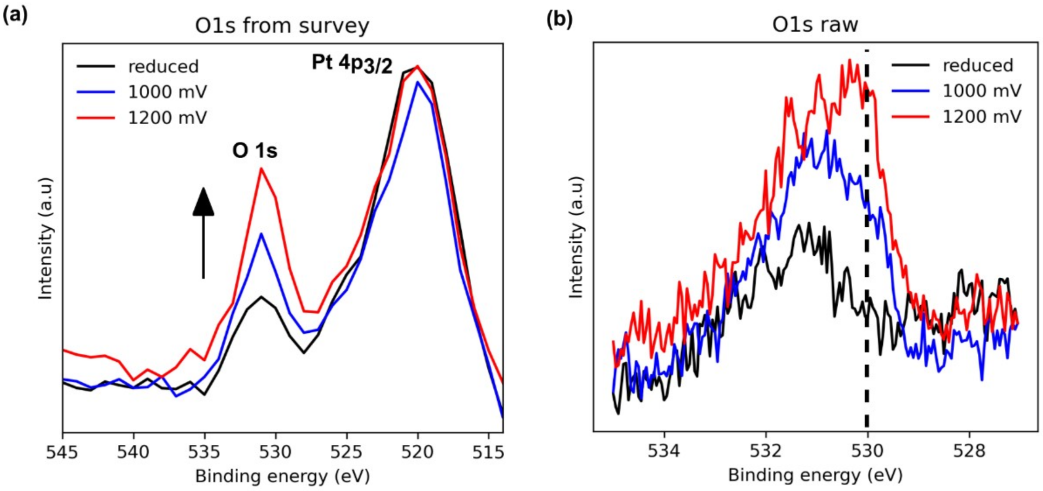

Figure S23: (a) Raw O 1s and Pt $4f_{3/2}$ from the survey spectra as a function of potential. (b) Background-subtracted O 1s spectra as a function of potential. The binding energy of metal oxide is marked.

Supporting electrochemical data

Figure S24 shows the CV cycles used to "activate" the polycrystalline platinum surface and reach a steady-state polycrystalline platinum voltammogram. Blue is the first cycle, and red is the last.

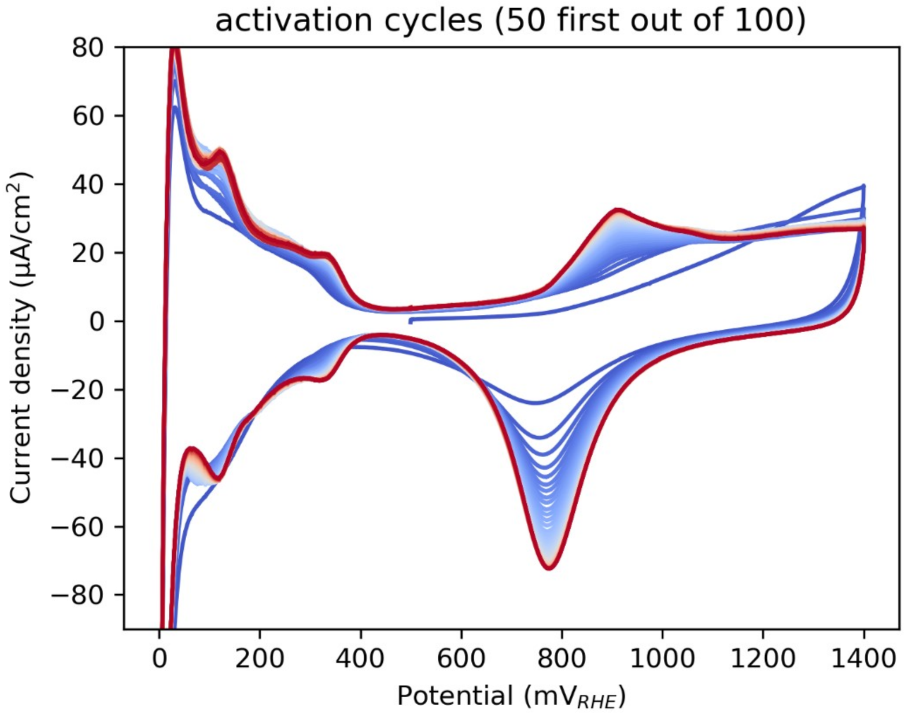

Figure S24: CVs during oxidation-reduction cycles to establish a steady-state polycrystalline platinum voltammogram.

Figure S25 shows blank voltammograms (0.1 M $HClO_4$, Ar saturated) for different upper vertex potentials measured for the platinum polycrystal mounted in the RDE sample holder in a glass cell. Figure S26 (a) shows the blank voltammograms as a function of scan rate, and (b) shows the background-subtracted anodic charge for the oxidation peak. Figure S27 shows the background subtraction of the linear sweep voltammogram of ORR as a function of scan rate. Figure S28 shows the blank cathodic linear sweep voltammogram after different lengths of hold time at 1 V.

Figure S29 shows cathodic LSVs in Ar and O_2-saturated conditions for various scan rates after potential holds at 1 V. As seen in (b), the onset of ORR shifts to lower potentials for higher scan rates, consistent with the shift in the reduction peak in (a). So, for a constant initial oxide coverage, generated by 100 s hold times at 1 V, the ORR onset shifts to higher overpotentials with higher scan rates. During CVs, the ORR onset shifts to lower overpotentials for higher scan rates during the cathodic sweep, as shown in Figure 3 in the main text.

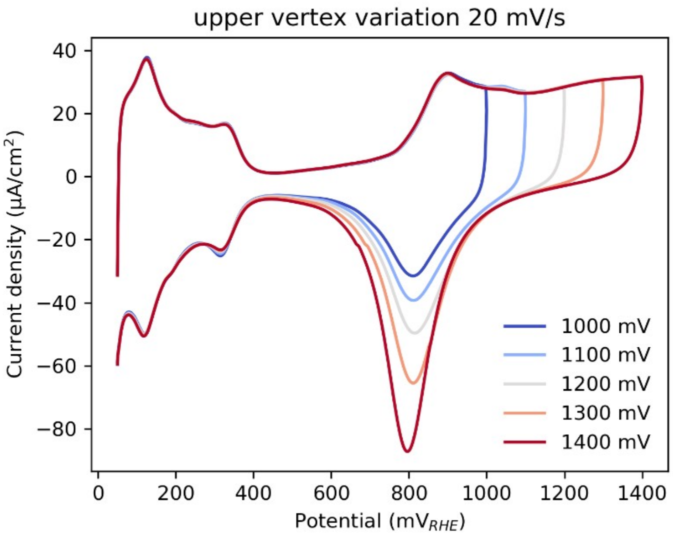

Figure S25: Blank voltammogram for different upper vertex potentials measured in 0.1 M $HClO_4$, Ar saturated, at 20 mV/s.

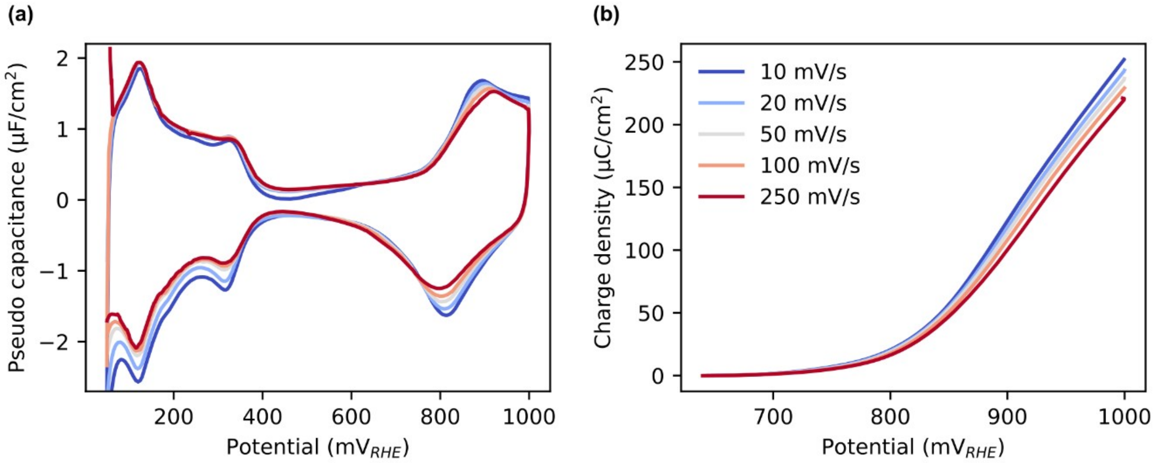

Figure S26: (a) Blank voltammogram at different scan rates measured in 0.1 M $HClO_4$, Ar saturated. (b) Integrated background-corrected anodic charge.

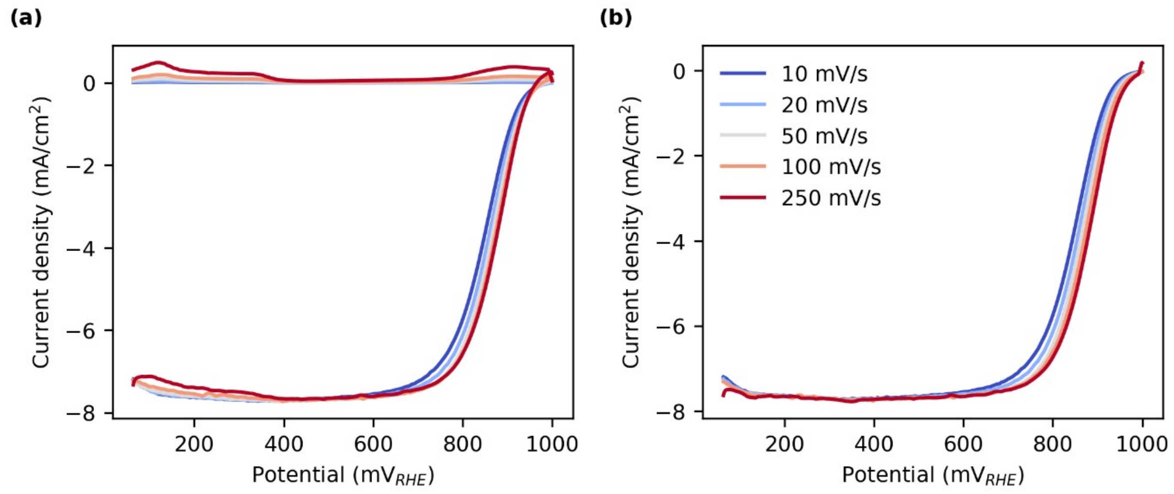

Figure S27: (a) Blank (Ar) and O_2 saturated linear sweep voltammograms in 0.1 M $HClO_4$ measured at 2500 rpm. (b) O_2 saturated voltammogram after subtracting the blank, as shown in Figure 3 in the main text.

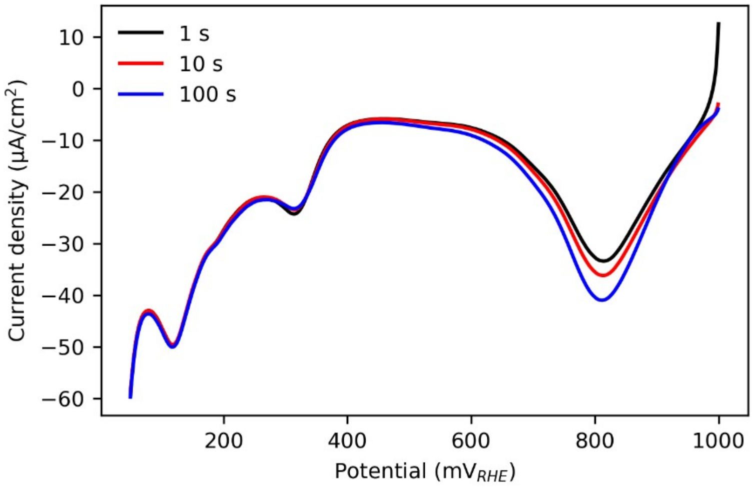

Figure S28: (a) Blank voltammogram after different hold times at 1 V in 0.1 M $HClO_4$, Ar-saturated, measured at 20 mV/s.

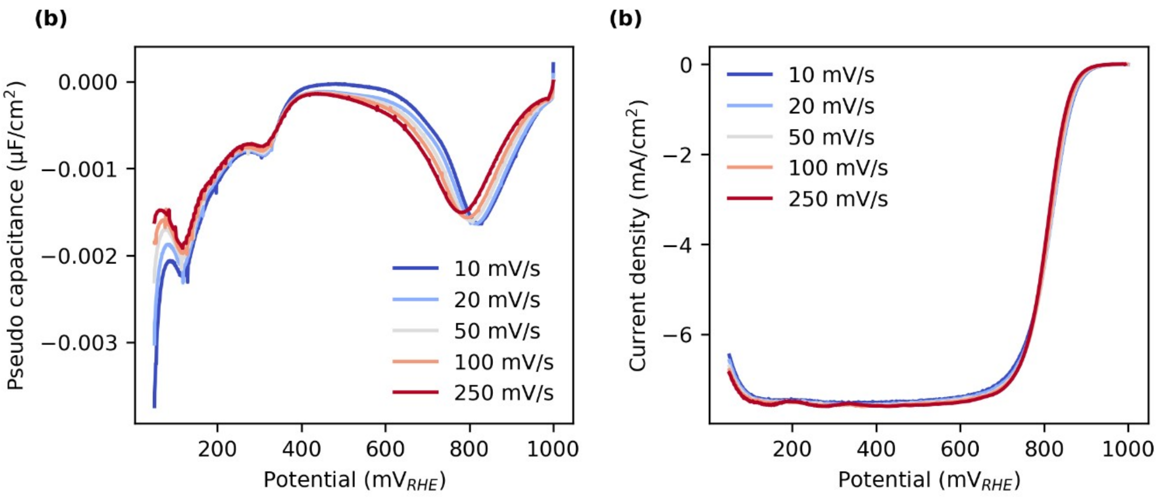

Figure S29: (a) Cathodic LSV after 100s hold time at 1 V for various scan rates measured in Ar-saturated 0.1 M $HClO_4$. (b) Cathodic LSV after 100s hold time at 1 V for various scan rates measured in O_2-saturated 0.1 M HClO4 at 2500 rpm. The Ar-saturated voltammograms have been subtracted to highlight only the current from oxygen reduction.

Microkinetic modeling

We have decided to create a kinetic model to better demonstrate the effect surface oxide formation has on ORR. The model we employed is a very simplified picture of the real surface condition that qualitatively shows the influence of oxide coverage on ORR, while also trying to capture general trends seen from the RefleXAFS. The model must thus satisfy the following: first, the oxide coverage must increase with increasing upper vertex potential, second, it must roughly capture the general trends of oxidation/reduction, and third, show the correct hysteresis between the positive and negative scan in the ORR.

To satisfy the self-imposed conditions, we developed our own model, which is different from what was made previously. A good overview of the different models employed over the years can be found in [19]. While other models correctly predict the shape and trends of electrochemical data, for both platinum oxidation and ORR, said models in general do not discuss how the two processes influence each other, with the paper of Jayasankar et. al being a notable exception. [19-25] However even in that paper Jayasankar et. al do not fully demonstrate the experimentally observed ORR hysteresis [19].

We based our model on the findings of Conway, where he assumes multiple sites that ultimately form the final oxide structure [26, 27]. Our model is created as a balance between making accurate qualitative and some quantitative predictions, while being formulated in the simplest way possible. Thus, it should be stated that the model details are of no great importance, as they do not represent a completely accurate picture on the surface during an experiment.

We have decided to model the platinum oxidation, as shown below, occurring on two different Pt sites (Pt^*_I and Pt^*_{II}). Initial OH adsorption on the two different sites is shown by equations 1 and 3, giving $Pt^*_I OH$ and $Pt^*_{II} OH$. Modeled by equations 2 and 4, the two sites place-

exchange with OH thus becoming one site ($OHPt^*$). The newly formed site is then further oxidized to form Pt^*O which can chemically transform into surface platinum oxide (PtO), which acts as a blocking species for ORR. These final oxidation stages are represented by equations 5 and 6. An important assumption is that such an oxidation path occurs irreversibly, thus in order to reduce the surface oxide it must proceed via a different pathway. This is represented with equation 7, where PtO reduces directly to a clean Pt^*_I. Here we do not have two distinct pathways, as that would complicate the model even further, without giving additional insights into the studied behavior. This is because we use a very simplified picture of the ORR, equation noted as ORR.

$$Pt^*_I + H_2O \rightleftharpoons Pt^*_I OH + H^+ + e^- \#(1)$$

$$Pt^*_I OH \rightarrow OHPt^* \#(2)$$

$$Pt^*_{II} + H_2O \rightleftharpoons Pt^*_{II} OH + H^+ + e^- \#(3)$$

$$Pt^*_{II} OH \rightarrow OHPt^* \#(4)$$

$$OHPt^* + H_2O \rightarrow Pt^*O + H^+ + e^- \#(5)$$

$$Pt^*O \rightleftharpoons PtO \#(6)$$

$$PtO + 2e^- + 2H^+ \rightleftharpoons Pt^*_I \#(7)$$

$$Pt^*_I + A_{ORR} + e^- \rightarrow Pt^*_I + B_{ORR} \#(ORR)$$

The full set of equations above creates a hysteresis, and alters the overpotential when changing the upper vertex potential and scan rate. We decided to simplify the matter by simulating the ORR as a simple 1 electron transfer reaction limited by mass transport. The conversion of A_{ORR} and B_{ORR}, which act as analogs for oxygen and water, can only occur if there are free platinum (Pt_I^*) sites present. Combining this with our version of the platinum oxidation model, we can successfully demonstrate how ORR is limited by site availability and not by the sluggishness of any ORR step. Parameters used in the model are shown in Table S3.

The model itself is a set of coupled ordinary (ODE) and partial differential equations (PDE). The ODEs constitute the oxidation mechanism, while two PDEs are then added to approximate the ORR behavior. The PDEs in this case are 2nd Fick's law, describing diffusion of the species to and from the electrode. The boundary at the electrode is treated by coupling the flux with an appropriate rate law. The ODEs on the other hand consist of interwoven rate laws. The potential dependence of the rate law is modelled by the Butler-Volmer equation. To account for interactions between the surface species, the Frumkin isotherm is added to the rate law. This is shown generally for a reaction involving electrons in equations S1a, and for a potential independent reaction in equation S1b.

$$\frac{d\Gamma_i}{dt} = -k_{0,i}\Gamma_i e^{-\alpha\frac{zF}{RT}\left(E_{app} - E^0\right)} e^{-g_i\Gamma_i} + k_{0,i}\Gamma_j e^{(1-\alpha)\frac{zF}{RT}\left(E_{app} - E^0_i\right)} e^{-g_j\Gamma_j} \#(S1a)$$

$$\frac{d\Gamma_i}{dt} = - k_{f,i}\Gamma_i e^{-g_i\Gamma_i} + k_{b,i}\Gamma_j e^{-g_j\Gamma_j}\#(S1b)$$

The parameters needed to describe the full system are thus the kinetic constant (k_0), the charge transfer coefficient (α), and the formal potential (E^0) for each electrochemical reaction. For every chemical reaction the forward (k_f) and backward (k_b) rate constant are needed. To account for the isotherm in the rate equation, each species is assigned a parameter g_i. This represents the interaction between the atoms of the same species, which further influences the rates. Other types of interspecies interactions are not included so that the model is as simple as possible, while still reproducing the general shape of the CV. The differential equations (S1a and S1b) themselves inform the program how the surface concentration (Γ) of each species changes with time. These parameters were obtained with a fit to experimental data, and are shown in Table S3. To describe the PDE fully, as given by ElectroKitty, the diffusion coefficient (D), the fluid viscosity (ν), and the initial concentration are needed (c), which are taken from literature and also shown in Table S3 [28]. For ElectroKitty to output the current, the electrode area (A) is needed as well, which is a known experimental parameter. The applied potential (E_{app}) depends on the experiment used and as such is constructed to be exactly as the one used in the experiment. The experiment itself could be different potential holds, CVs with different vertex potentials or scan-rates. The constructed potential program must contain the time and potential points for which ElectroKitty then simulates the corresponding current.

Table S3: Parameters used in microkinetic modeling

**Parameter**	**Value**	**Parameter**	**Value**
α_1 [/]	1,561E-01	α_{ORR} [/]	5,000E-01
$k_{0,1}$ [1/s]	3,269E-03	$k_{0,ORR}$ [m^2/s]	2.500E+00
$E^{0'}_1$ [V]	1,649E+00	$E^{0'}_{ORR}$ [V]	1,050E+00
k_2 [1/s]	2,055E+03	Γ_{PtI*} [mol/m^3]	1,703E-05
α_3 [/]	4,136E-01	Γ_{PtII*} [mol/m3]	3,511E-05
$k_{0,3}$ [1/s]	3,579E-01	$g_{Ptoxide*}$ [/]	-9,647E+00
$E^{0'}_3$ [V]	8,483E-01	g_{OPt*} [/]	-2,998E+01
k_4 [1/s]	2,721E-02	g_{OHPt*} [/]	-2,388E+01
α_5 [/]	3,081E-01	g_{Pt1OH*} [/]	-6,366E+00
$k_{0,5}$ [1/s]	6,911E-02	g_{Pt1*} [/]	-4,299E+00
$E^{0'}_5$ [V]	4,997E-01	g_{PtOH*} [/]	2,839E+00
k_{6f} [1/s]	6,160E+03	g_{Pt*} [/]	-2,586E+01
k_{6b} [1/s]	1,105E+03	D [m^2/s]	3,500E-09
α_7 [/]	5,575E-01	c_{O2} [mol/l]	4.850E-03
$k_{0,7}$ [1/s]	1,752E-01	A [cm^2]	2,830E-01
$E^{0'}_7$ [V]	7,827E-01	ν [m^2/s]	1.000E-09

Supporting figures

Figure S30 shows a blank modelled voltammogram and the corresponding coverages. Figure S31 shows modelled blank voltammograms as a function of upper vertex potential and the corresponding oxide coverage. Figure S32 shows modelled blank voltammograms as a function

of scan rate and the corresponding oxide coverage. Figure S33 shows coverages during potential hold at 1 V. To obtain the surface coverages in Figure 5 of the main text and Figure S30 b, the concentrations containing the species were summed together. For example, our model supposes two Pt surface sites (Pt_I and Pt_{II}), these were summed together and represented as bare surface Pt, as any other surface sensitive technique we used would not be able to distinguish between the two.

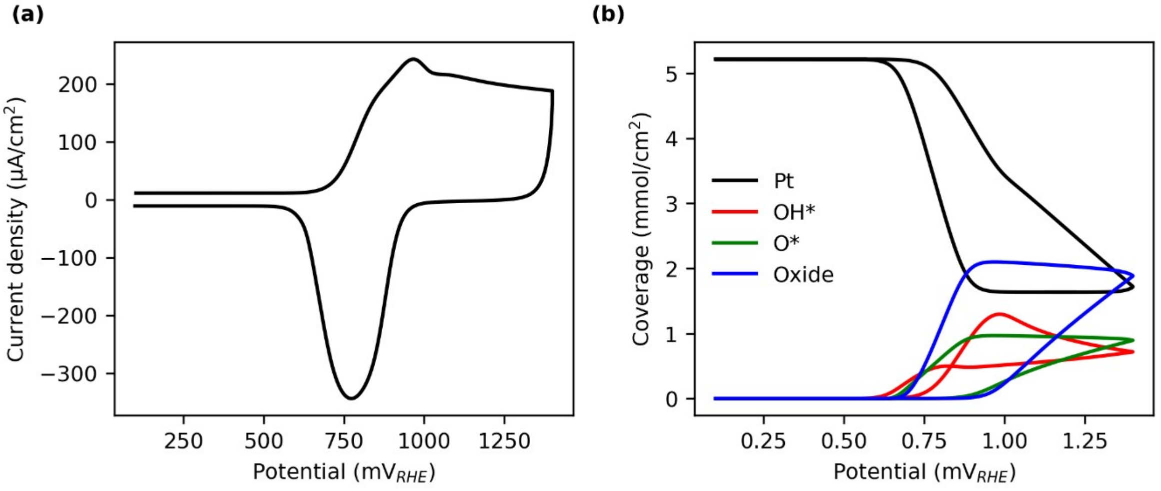

Figure S30: (a) Modeled blank CV at 100 mV/s using microkinetic modeling. (b) Corresponding coverages extracted from the microkinetic model.

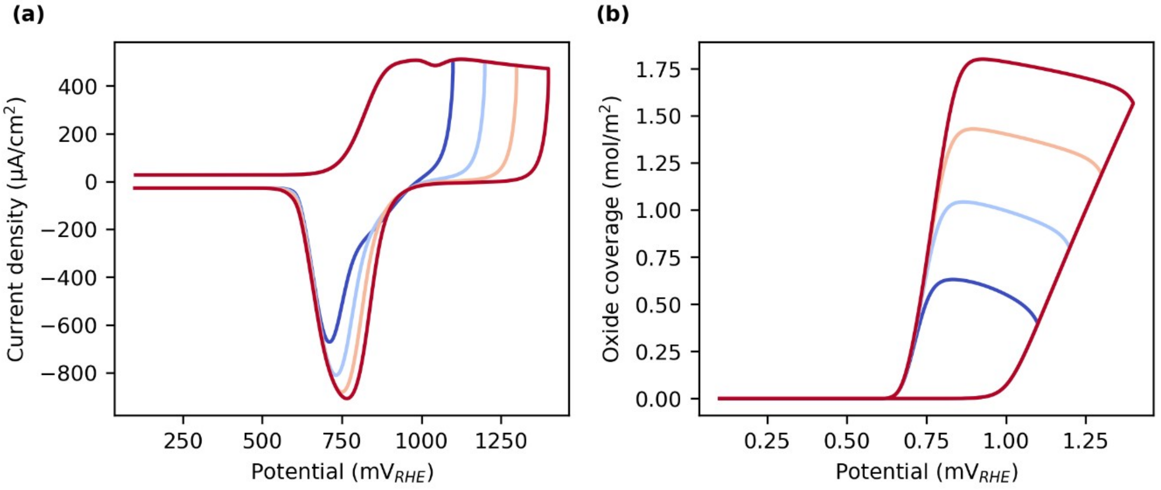

Figure S31: (a) Modeled CVs as a function of upper vertex potential at 250 mV/s. (b) Corresponding oxide coverages.

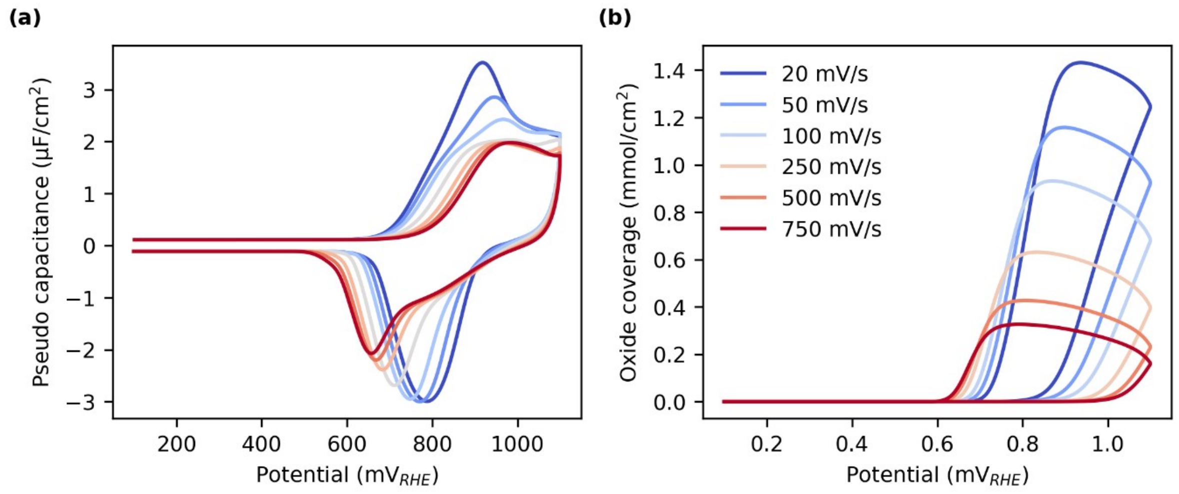

Figure S32: (a) Modeled CVs as a function of scan rate shown as pseudo-capacitance normalized by scan rate. (b) Corresponding oxide coverages.

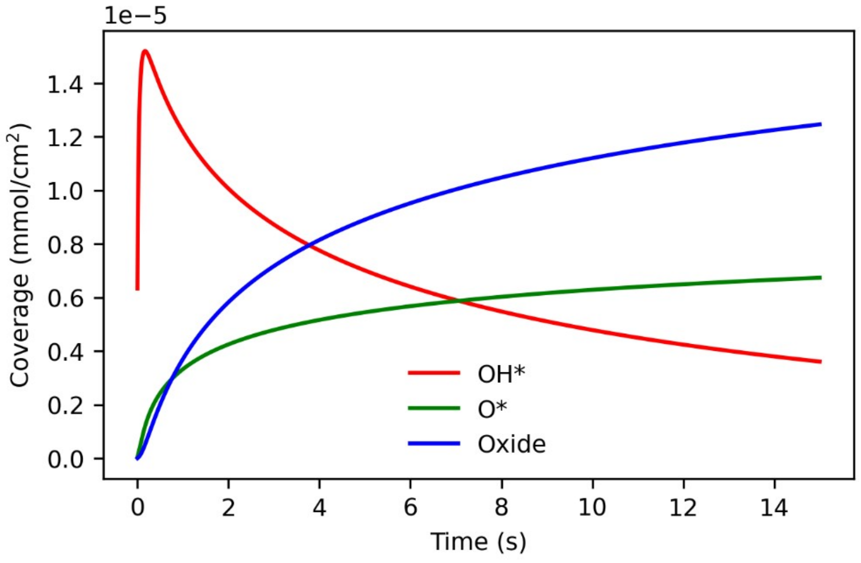

Figure S33: Modeled coverages during potential holds as used in Figure 5e in the main text.

References

1. Drnec, J., M. Ruge, F. Reikowski, B. Rahn, F. Carlà, R. Felici, J. Stettner, O.M. Magnussen, and D.A. Harrington, *Initial stages of Pt(111) electrooxidation: dynamic and structural studies by surface X-ray diffraction.* Electrochimica Acta, 2017. **224**: p. 220-227.
2. Henke, B.L., E.M. Gullikson, and J.C. Davis, *X-Ray Interactions: Photoabsorption, Scattering, Transmission, and Reflection at E = 50-30,000 eV, Z = 1-92.* Atomic Data and Nuclear Data Tables, 1993. **54**(2): p. 181-342.
3. Newville, M., *Larch: An Analysis Package for XAFS and Related Spectroscopies.* Journal of Physics: Conference Series, 2013. **430**(1): p. 012007.
4. Lützenkirchen-Hecht, D. and R. Frahm, *Structure of reactively sputter deposited tin-nitride thin films: A combined X-ray photoelectron spectroscopy, in situ X-ray reflectivity and X-ray absorption spectroscopy study.* Thin Solid Films, 2005. **493**(1): p. 67-76.

5. Bondarenko, A.S., I.E.L. Stephens, H.A. Hansen, F.J. Pérez-Alonso, V. Tripkovic, T.P. Johansson, J. Rossmeisl, J.K. Nørskov, and I. Chorkendorff, *The Pt(111)/Electrolyte Interface under Oxygen Reduction Reaction Conditions: An Electrochemical Impedance Spectroscopy Study.* Langmuir, 2011. **27**(5): p. 2058-2066.
6. Drnec, J., M. Ruge, F. Reikowski, B. Rahn, F. Carlà, R. Felici, J. Stettner, O.M. Magnussen, and D.A. Harrington, *Pt oxide and oxygen reduction at Pt(111) studied by surface X-ray diffraction.* Electrochemistry Communications, 2017. **84**: p. 50-52.
7. Kresse, G. and J. Hafner, *Ab initio molecular dynamics for liquid metals.* Physical review B, 1993. **47**(1): p. 558-561.
8. Kresse, G. and J. Hafner, *Ab initio molecular dynamics for open-shell transition metals.* Physical review B, 1993. **48**(17): p. 13115-13118.
9. Kresse, G. and J. Hafner, *Ab initio molecular-dynamics simulation of the liquid-metal--amorphous-semiconductor transition in germanium.* Physical review B, 1994. **49**(20): p. 14251-14269.
10. Kresse, G. and J. Furthmüller, *Efficient iterative schemes for ab initio total-energy calculations using a plane-wave basis set.* Physical review B, 1996. **54**(16): p. 11169-11186.
11. Perdew, J.P., K. Burke, and M. Ernzerhof, *Generalized Gradient Approximation Made Simple.* Physical Review Letters, 1996. **77**(18): p. 3865-3868.
12. Blöchl, P.E., *Projector augmented-wave method.* Physical review B, 1994. **50**(24): p. 17953-17979.
13. Kresse, G. and D. Joubert, *From ultrasoft pseudopotentials to the projector augmented-wave method.* Physical review B, 1999. **59**(3): p. 1758-1775.
14. Pehlke, E. and M. Scheffler, *Evidence for site-sensitive screening of core holes at the Si and Ge (001) surface.* Physical Review Letters, 1993. **71**(14): p. 2338-2341.
15. Newville, M., T. Stensitzki, D.B. Allen, and A. Ingargiola, *LMFIT: Non-Linear Least-Square Minimization and Curve-Fitting for Python.* 2014.
16. Schmid, M., H.-P. Steinrück, and J.M. Gottfried, *A new asymmetric Pseudo-Voigt function for more efficient fitting of XPS lines.* Surface and Interface Analysis, 2014. **46**(8): p. 505-511.
17. Shirley, D.A., *High-Resolution X-Ray Photoemission Spectrum of the Valence Bands of Gold.* Physical Review B, 1972. **5**(12): p. 4709-4714.
18. Tanuma, S., C.J. Powell, and D.R. Penn, *Calculations of electron inelastic mean free paths. V. Data for 14 organic compounds over the 50–2000 eV range.* 1994. **21**(3): p. 165-176.
19. Jayasankar, B. and K. Karan, *O2 electrochemistry on Pt: A unified multi-step model for oxygen reduction and oxide growth.* Electrochimica Acta, 2018. **273**: p. 367-378.
20. Fukaya, N., H. Murata, M. Shibata, and R. Jinnouchi, *Micro-kinetic mean-field model of subsurface oxidation in a platinum electrocatalyst.* Electrochimica Acta, 2023. **464**: p. 142867.
21. Parthasarathy, A., S. Srinivasan, A.J. Appleby, and C.R. Martin, *Pressure Dependence of the Oxygen Reduction Reaction at the Platinum Microelectrode/Nafion Interface: Electrode Kinetics and Mass Transport.* Journal of the Electrochemical Society, 1992. **139**(10): p. 2856.
22. Moore, M., A. Putz, and M. Secanell, *Investigation of the ORR Using the Double-Trap Intrinsic Kinetic Model.* Journal of the Electrochemical Society, 2013. **160**(6): p. F670.
23. Appleby, A.J., *Theory of Successive Electron Transfer Steps in Cyclic Voltammetry: Application to Oxygen Pseudocapacitance on Platinum.* Journal of the Electrochemical Society, 1973. **120**(9): p. 1205.
24. Jayasankar, B.r., K. Karan, and D.B. Harvey, *Multi-Step Oxygen Reduction Reaction (ORR) Kinetics on Pt including Water Activation.* ECS Meeting Abstracts, 2012. **MA2012-02**(13): p. 1570.
25. Wang, J.X., J. Zhang, and R.R. Adzic, *Double-Trap Kinetic Equation for the Oxygen Reduction Reaction on Pt(111) in Acidic Media.* The Journal of Physical Chemistry A, 2007. **111**(49): p. 12702-12710.
26. Angerstein-Kozlowska, H., B.E. Conway, and W.B.A. Sharp, *The real condition of electrochemically oxidized platinum surfaces: Part I. Resolution of component processes.* Journal of Electroanalytical Chemistry and Interfacial Electrochemistry, 1973. **43**(1): p. 9-36.
27. Conway, B.E. and R. Greef, *Theory and Principles of Electrode Processes.* Journal of the Electrochemical Society, 1966. **113**(12): p. 325C.

28. B. E. . Poling, J.M.P., and J. P. . O'Connell, *Properties of Gases and Liquids, Fifth Edition.* McGraw-Hill Education, 2020.
